# Serum YB-1 links dyslipidemia to NET-mediated vascular calcification in hemodialysis

**DOI:** 10.1186/s12944-025-02832-y

**Published:** 2026-01-13

**Authors:** Jiaxin Chen, Li Wang, Shuan Zhao, Jian Zhang, Nana Song, Yi Fang, Zhen Zhang, Xuesen Cao, Bo Shen, Jie Teng, Jianzhou Zou, Jieru Cai, Xiaoqiang Ding, Jialin Wang

**Affiliations:** 1 Department of Nephrology, Zhongshan Hospital, Fudan University, Fenglin Road 180, Shanghai, 200032 China; 2 Department of Nephrology, Zhongshan Hospital (Xiamen), Fudan University, Fujian, 361016 China

**Keywords:** Y-Box-Binding protein 1, Vascular calcification, Hemodialysis, Neutrophil extracellular traps, Dyslipidemias

## Abstract

**Background:**

Vascular calcification (VC) is highly prevalent in patients undergoing maintenance hemodialysis (MHD) and is associated with cardiovascular morbidity. However, traditional lipid and mineral markers have limited predictive value. Y-box binding protein-1 (YB-1), a regulator of lipid metabolism and inflammation, may provide additional mechanistic and clinical insight.

**Methods:**

Serum YB-1 was measured in 209 MHD patients (30-month follow-up) who were stratified into hyperlipidemia and control groups. Receiver Operating Characteristic (ROC) and decision curve analysis (DCA, threshold range 0.1–0.4) were used to assess the predictive performance of YB-1 against traditional models based on lipid, glucose, and bone metabolism. Mechanistic studies in HL-60-derived neutrophil-like cells and a 5/6 nephrectomized rat model were performed to assess the role of YB-1 in neutrophil extracellular trap (NET) formation and VC.

**Results:**

Serum YB-1 was significantly elevated in hyperlipidemia patients and was independently associated with new-onset VC (AUC 0.707, 95% CI 0.630–0.784). YB-1 outperformed lipid-, glucose-, and bone-based models, providing added net clinical benefit in DCA within the 0.24–0.33 threshold range. Mechanistically, serum levels of citrullinated histone H3 (citH3), a NET marker, were increased in hyperlipidemia patients. In vitro, YB-1 and IS synergistically enhanced neutrophil lipid droplet accumulation and citH3 release, while NET-rich supernatants promoted VSMC calcification. In vivo, IS-treated 5/6 nephrectomy rats displayed elevated YB-1, increased citH3, and aggravated aortic calcification.

**Conclusion:**

Serum YB-1 is a novel predictor and potential mechanistic mediator of VC in MHD patients. Incorporating YB-1 into existing clinical risk models may support earlier recognition of individuals at elevated cardiovascular risk and inform more effective management strategies to improve long-term health outcomes.

**Supplementary Information:**

The online version contains supplementary material available at 10.1186/s12944-025-02832-y.

## Highlights

Extracellular YB-1 reprograms neutrophil lipid metabolism to enhance NET formation and drive VSMC calcification under uremic conditions.

Clinically, serum YB-1 levels are independently associated with dyslipidemia in MHD patients.

YB-1 acts as a mechanistic bridge linking dyslipidemia to vascular calcification in MHD patients.

Integrating YB-1 improves vascular calcification prediction beyond traditional metabolic markers, providing measurable net clinical benefit in intermediate-risk MHD patients.

**Supplementary Information:**

The online version contains supplementary material available at 10.1186/s12944-025-02832-y.

## Introduction

Vascular calcification (VC) is a key contributor to cardiovascular morbidity and mortality in patients with end-stage renal disease (ESRD), particularly among patients undergoing maintenance hemodialysis (MHD) [1]. Although dyslipidemia contributes significantly to the risk of cardiovascular risk in the general population, its association with outcomes in dialysis patients is paradoxical (“reverse epidemiology”) [2, 3], and statin trials have largely failed to demonstrate significant cardiovascular benefits in this setting [4, 5]. These observations question the adequacy of traditional lipid markers for risk stratification in MHD.

Beyond lipid abnormalities, uremic toxins, chronic inflammation, and immune dysregulation shape the cardiovascular landscape in ESRD [6, 7]. Neutrophil extracellular traps (NETs) have been recognized as critical drivers of vascular injury and calcification [8, 9]. Y-box binding protein-1 (YB-1) is a multifunctional cold shock protein implicated in lipid metabolism and inflammatory signaling [10–12] and has been recognized as a constituent of NETs [13]. Whether YB-1 functionally links dyslipidemia to NET-driven vascular injury and whether it improves VC risk prediction in MHD remain unclear.

This study sought to clarify both the clinical predictive value and mechanistic role of YB-1 in MHD patients, hypothesizing that it functions not merely as a circulating biomarker but also as an active mediator that promotes vascular injury through neutrophil lipid reprogramming and NET formation, ultimately driving VC. To test this, a retrospective clinical cohort was combined with mechanistic in vitro experiments and an in vivo 5/6 nephrectomy rat model. This integrated approach extends previous descriptive findings toward establishing a causal and translational link between YB-1 activation, dyslipidemia, and VC in MHD patients.

## Methods

### Cohort description and study design

**Fig. 1:**
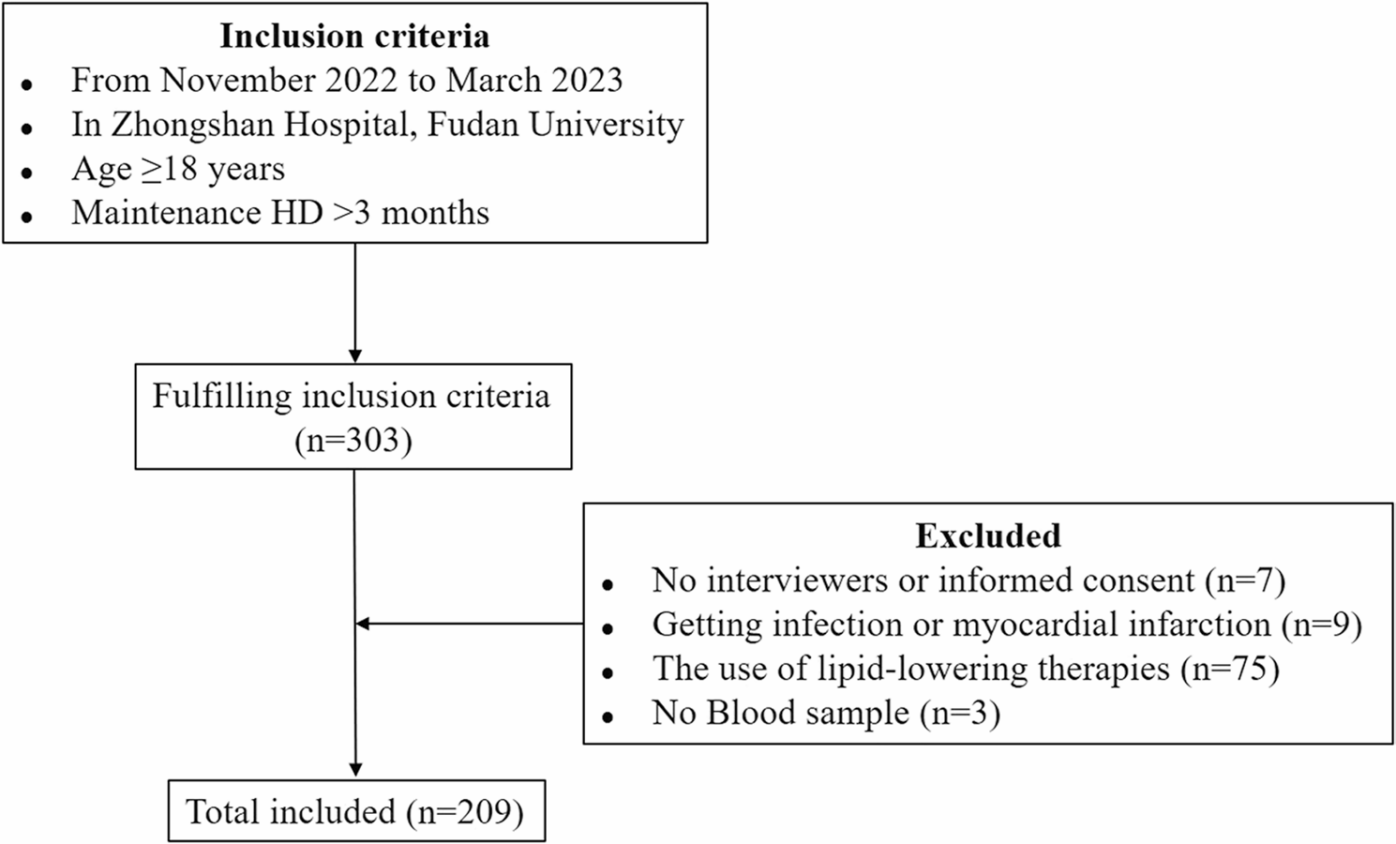
Flowchart of participant enrollment. Among a cohort of 303 patients undergoing MHD, 209 individuals who met the inclusion and exclusion criteria were enrolled in the final analysis

This retrospective cohort study enrolled adult patients (> 18 years) undergoing MHD at Zhongshan Hospital, Fudan University, between November 2022 and March 2023. Inclusion criteria required patients to be on regular dialysis for at least 3 months. Exclusion criteria included recent infection or myocardial infarction (< 1 month), use of lipid-lowering medications, inability to provide informed consent, or missing serum samples. A total of 209 eligible patients were included (Fig. 1). Patients were stratified into a hyperlipidemia (HLP) group (total cholesterol > 5.2 mmol/L and/or triglycerides > 1.7 mmol/L) and a control (CON) group in accordance with the 2016 Chinese Guidelines for the Management of Dyslipidemia in Adults [14]. Baseline demographics, comorbidities, dialysis vintage, and laboratory parameters (including full lipid panel, glucose/HbA1c, and mineral metabolism indices) were compared between HLP and CON groups. Variables that showed potential imbalance (*P* < 0.05) were subsequently adjusted for in multivariable analyses to minimize confounding. Because of the limited sample size, formal propensity matching was not performed; instead, adjusted regression models were used to ensure comparability between groups. VC was assessed by coronary computed tomography angiography and carotid ultrasound, and incident VC events were prospectively recorded during a 30-month follow-up period. This study was approved by the Institutional Review Board of Zhongshan Hospital (approval number: B2021-740) and conducted in accordance with the Declaration of Helsinki, with all participants providing written informed consent.

### Laboratory measurement

Blood samples were obtained pre-dialysis from arterial vascular access. Serum was separated by centrifugation and stored at -80 °C. YB-1 levels were quantified by a commercially available ELISA kit (LifeSpan BioSciences, WA, USA) following the manufacturer’s instructions. Lipid profiles (total cholesterol, triglycerides, LDL-C, HDL-c), creatinine, phosphate, calcium, high-sensitivity C-reactive protein (hs-CRP), and complete blood counts were measured by standardized clinical laboratory protocols in the Department of Laboratory Medicine at Zhongshan Hospital.

Peripheral lymphocyte subsets (CD3+, CD4+, CD8+, CD19+, and NK cells) were analyzed using flow cytometry (BD Biosciences, USA) [15]. Serum levels of cytokines (tumor necrosis factor (TNF)-a, interleukin (IL)-1b, IL-2R, IL-6, and IL-8) were quantified by ELISA (R&D systems, USA) according to the manufacturer’s protocols. All samples were analyzed in duplicate.

### In vitro neutrophil stimulation and lipid metabolism assay

HL-60 promyelocytic leukemia cells (ATCC, USA) were cultured in RPMI 1640 with 10% FBS. Neutrophil differentiation was induced using 1mM retinoic acid (RA; Sigma-Aldrich, St Louis, MO, USA) for 72 h. Differentiated cells were treated with 1 mM indoxyl sulfate (IS) (Sigma-Aldrich) and 100 ng/ml recombinant YB-1 (rYB-1; Abnova, Taiwan, China) for another 3 h.

Lipid metabolism-related genes (Hilpda, Srebp2, Soat1, ABCA1, and PGC1a) was analyzed by qRT-PCR. Protein levels (YB-1 and citrullinated histone H3(citH3)) were assessed by Western blot. Lipid droplets were visualized with BODIPY 493/503 staining (Beyotime Biotechnology, Shanghai, China) according to the manufacturer’s instructions.

### NETs-VSMC calcification assay

To assess the functional consequences of neutrophil activation, cell culture supernatants were collected from HL-60 cells treated with IS and rYB-1. These NETs-rich supernatants were combined with vascular smooth muscle cells (VSMCs) culture medium at a 2:1 ratio and co-incubated with human VSMCs for 72 h in osteogenic medium. VSMC calcification was assessed using Alizarin Red S staining.

### Animal model of uremia and vascular calcification

Male Sprague-Dawley rats (180–220 g) were purchased from SLAC Laboratory animal Co. LTD, Shanghai, China. The 5/6 nephrectomy (5/6 Nx) operation was performed to induce chronic kidney disease (CKD). Four weeks after surgery, rats were randomly assigned to receive IS (100 mg/kg, twice a week, intraperitoneally) or saline alone as control for 14 weeks.

At endpoint, serum samples were analyzed for YB-1, lipid profiles, creatinine, and citH3. Aortas were harvested and subjected to Alizarin Red staining to evaluate VC. VC-related genes (RUNX2, BMP2, BGLAP, and ALPL) was analyzed by qRT-PCR. Experimental rats were humanely sacrificed by decapitation. All experimental protocols received approval from the Institutional Animal Care and Use Committee of Fudan University (Approval No. 2023 − 109) and adhered strictly to the National Institutes of Health Guide for laboratory animal care. Measures were implemented throughout the study to reduce animal suffering to the greatest extent possible.

### Statistical analysis

Continuous variables were expressed as mean ± standard deviation (SD) or median (interquartile range, IQR) as appropriate, and categorical variables as counts and percentages. Statistical comparisons between the HLP and CON groups were performed using either Student’s t-test for continuous variables or the chi-square test for categorical variables, depending on the type of data. The analysis of variance followed by Tukey’s *post-hoc* test was used for comparisons involving more than two groups. Variables demonstrating significant differences between the two groups in the baseline analysis were entered into a binary logistic regression model to determine independent predictors of hyperlipidemia. This study employed a classical maximum-likelihood binary logistic regression model as implemented in SPSS. This modelling approach does not incorporate any form of regularisation (e.g., L1 or L2). Consequently, standardisation of continuous predictors was not required. All continuous variables were retained on their original scales to preserve the interpretability and clinical relevance of the regression coefficients.

Correlations were performed to assess associations between serum YB-1 and other parameters by Spearman’s rank analysis. To evaluate the predictive ability of the models, predicted probabilities for each observation were generated based on the fitted model. The resulting predicted probabilities were subsequently utilized to generate receiver operating characteristic (ROC) curves. The predictive performance of serum YB-1 for VC was evaluated separately using ROC curve analysis, with the area under the curve (AUC) compared among composite models based on lipid-, glucose-, and bone-metabolism parameters. Decision curve analysis (DCA) was additionally conducted to evaluate the net clinical benefit of YB-1 and multivariable models across a range of clinically plausible decision thresholds. All statistical analyses were performed using SPSS version 26.0 (IBM Corp., Armonk, NY, USA), GraphPad Prism 8.0 (GraphPad Software, San Diego, CA, USA) and R version 4.4.2 (R Foundation for Statistical Computing, Vienna, Austria), with two-tailed *P* < 0.05 considered statistically significant. All in vitro experiments were conducted at least in triplicates.

## Results

### Baseline characteristics

Of 209 MHD patients (64.1% male; mean age 59.7 years), 97 (46.4%) were classified as HLP and 112 (53.6%) as CON (Table 1). Compared with controls, the HLP group exhibited significantly higher levels of HbA1c (6.0 ± 1.2% vs. 5.6 ± 0.8%, respectively; *P* = 0.005) and phosphate (2.4 ± 0.7 vs. 2.3 ± 0.6 mmol/L, respectively; *P* = 0.029), along with lower levels of 25(OH)D3 (29.0 ± 11.1 vs. 35.1 ± 16.4 nmol/L, respectively; *P* = 0.002) (Tables 1 and 2). No substantial variations were observed in inflammatory and cardiovascular parameters.

**Table 1:** Clinical baseline characteristics of HD patients with and without hyperlipidemia

Variables	Overall (*n* = 209)	CON (*n* = 112)	HLP (*n* = 97)	*P* value
Age, y	59.7 ± 15.2	60.3 ± 15.6	59.0 ± 14.8	0.533
Male, *N* (%)	134 (64.1%)	76 (67.8%)	58 (59.8%)	0.226
Diabetes, *N* (%)	47 (22.5%)	23 (20.5%)	24 (24.7%)	0.468
Hypertension, *N* (%)	184 (88.0%)	99 (88.4%)	85 (87.6%)	0.865
Cause of ESRD				
Primary glomerulonephritis, *N* (%)	109 (52.2%)	55 (49.1%)	54 (55.7%)	0.344
Diabetic nephropathy, *N* (%)	31 (14.8%)	18 (16.0%)	13 (13.4%)	0.588
Hypertensive nephropathy, *N* (%)	20 (9.6%)	11 (9.8%)	9 (9.3%)	0.894
Polycystic nephropathy, *N* (%)	19 (9.1%)	11 (9.8%)	8 (8.2%)	0.693
Tumor, *N* (%)	3 (1.4%)	2 (1.8%)	1 (1.0%)	1.000
Obstructive uropathy, *N* (%)	5 (2.4%)	4 (3.6%)	1 (1.0%)	0.456
unknown, *N* (%)	30 (14.4%)	11 (9.8%)	11 (11.3%)	0.721
Dialysis vintage, y	6.1 ± 4.7	5.8 ± 4.6	6.3 ± 4.8	0.443
spKt/V	1.3 ± 0.5	1.3 ± 0.6	1.2 ± 0.2	0.068
30-month follow-up				
All-cause death, *N* (%)	36 (17.2%)	18 (16.1%)	18 (18.6%)	0.635
New vascular calcification, *N* (%)	60 (28.7%)	24 (21.4%)	36 (37.1%)	0.012*

*ESRD *end-stage renal disease; the data in the table are expressed as mean ± standard deviation or number (%); *P < 0.05

**Table 2:** Biological baseline characteristics of HD patients with and without hyperlipidemia

Variables	Overall (*n* = 209)	CON (*n* = 112)	HLP (*n* = 97)	*P* value
YB-1, ng/ml	1.1 ± 0.8	1.0 ± 0.7	1.2 ± 0.8	0.023*
C reactive protein, mg/L	4.0 (1.2, 9.3)	4.1 (1.2, 9.2)	4.0 (1.2, 9.2)	0.696
PLT, 1 × 10^9/L	184.4 ± 64.9	176.5 ± 62.3	193.6 ± 66.8	0.056
Anemia				
Hemoglobin, g/L	117.0 ± 17.5	115.1 ± 19.5	119.1 ± 14.8	0.105
Ferritin, ng/ml	177.0 (103.5, 352.5)	176.0 (104.5, 351.5)	178.0 (106.5, 357.5)	0.335
Transferrin saturation, %	26.0 (18.0, 38.0)	26.0 (18.5, 37.0)	26.0 (19.0, 37.5)	0.736
Albumin, g/L	39.3 ± 3.5	39.1 ± 3.6	39.5 ± 3.3	0.379
HbA1c, %	5.8 ± 1.0	5.6 ± 0.8	6.0 ± 1.2	0.005**
Glucose, mmol/L	7.4 ± 3.3	7.0 ± 2.4	7.8 ± 4.1	0.090
Bone metabolism				
Calcium, mmol/L	2.3 ± 0.2	2.3 ± 0.2	2.3 ± 0.2	0.950
Phosphate, mmol/L	2.3 ± 0.7	2.3 ± 0.6	2.4 ± 0.7	0.029*
iPTH, pg/ml	271.5 (166.5, 399.5)	275.0 (167.5, 400.5)	271.5 (167.5, 400.5)	0.494
25(OH)D, nmol/L	32.3 ± 14.5	35.1 ± 16.4	29.0 ± 11.1	0.002**
Cardiovascular parameters				
cTnT, *10^-2ng/ml	5.9 (4.1, 9.6)	5.9 (4.2,9.6)	5.9 (4.2, 9.6)	0.191
LVEF, %	61.7 ± 8.0	61.2 ± 8.5	62.4 ± 7.4	0.279
Vascular calcification, *N* (%)	85 (40.7%)	39 (34.8%)	46 (47.4%)	0.157
CIMT, mm	0.8 ± 0.2	0.8 ± 0.2	0.9 ± 0.2	0.415
Carotid artery plaque, *N* (%)	95 (45.5%)	47 (42.0%)	48 (49.5%)	0.335

*LVEF* left ventricular ejection fraction, *CIMT* carotid intima-media thickness; the data in the table are expressed as mean ± standard deviation or number (%); *P < 0.05; **P < 0.01

**Fig. 2:**
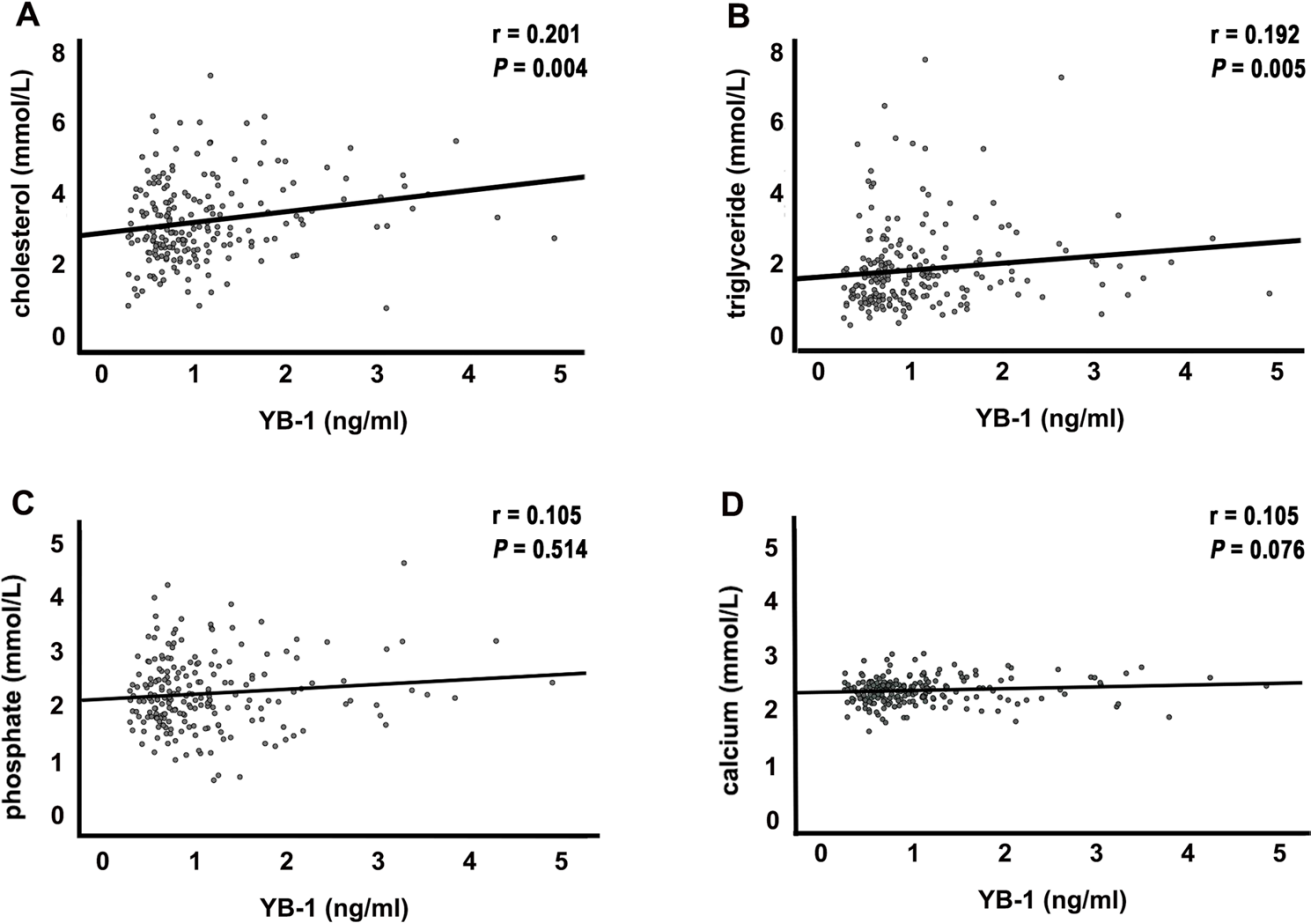
Correlation between serum YB-1 and metabolic parameters in MHD patients (*n* = 209). Scatter plots showing correlation analysis between serum YB-1 and total cholesterol **A**, triglycerides **B**, serum phosphorus (**C**), and serum calcium **D.** YB-1, Y-box-binding protein-1

Serum YB-1 concentrations were markedly elevated in the HLP group relative to the CON group (1.22 ± 0.81 vs. 0.98 ± 0.72 ng/ml, respectively; *P* = 0.023). Correlation analysis indicated a positive association between serum YB-1 and both total cholesterol (*r* = 0.201, *P* = 0.004; Fig. 2a) and triglyceride (*r* = 0.192, *P* = 0.005; Fig. 2b). In multivariate logistic regression analysis adjusted for HbA1c, phosphate, and 25(OH)D3, serum YB-1 remained an independent predictor of dyslipidemia (*P* = 0.01) (Table 3).

**Table 3:** Binary logistics regression analysis to evaluate the relation between independent variables and hyperlipidemia in HD patients (*N* = 209)

Variables	b-coefficient	SE	Wald	OR	95% CI	*P* value
YB-1	0.545	0.213	6.575	1.725	1.137–2.616	0.010*
HbA1c	0.480	0.164	8.624	1.616	1.173–2.227	0.003**
Phosphate	0.415	0.244	2.885	1.514	0.938–2.444	0.089
25(OH)D3	-0.031	0.013	6.307	0.969	0.946–0.993	0.012

*P < 0.05; **P < 0.01

### Serum YB-1 predicts the risk of vascular calcification in MHD patients

**Fig. 3:**
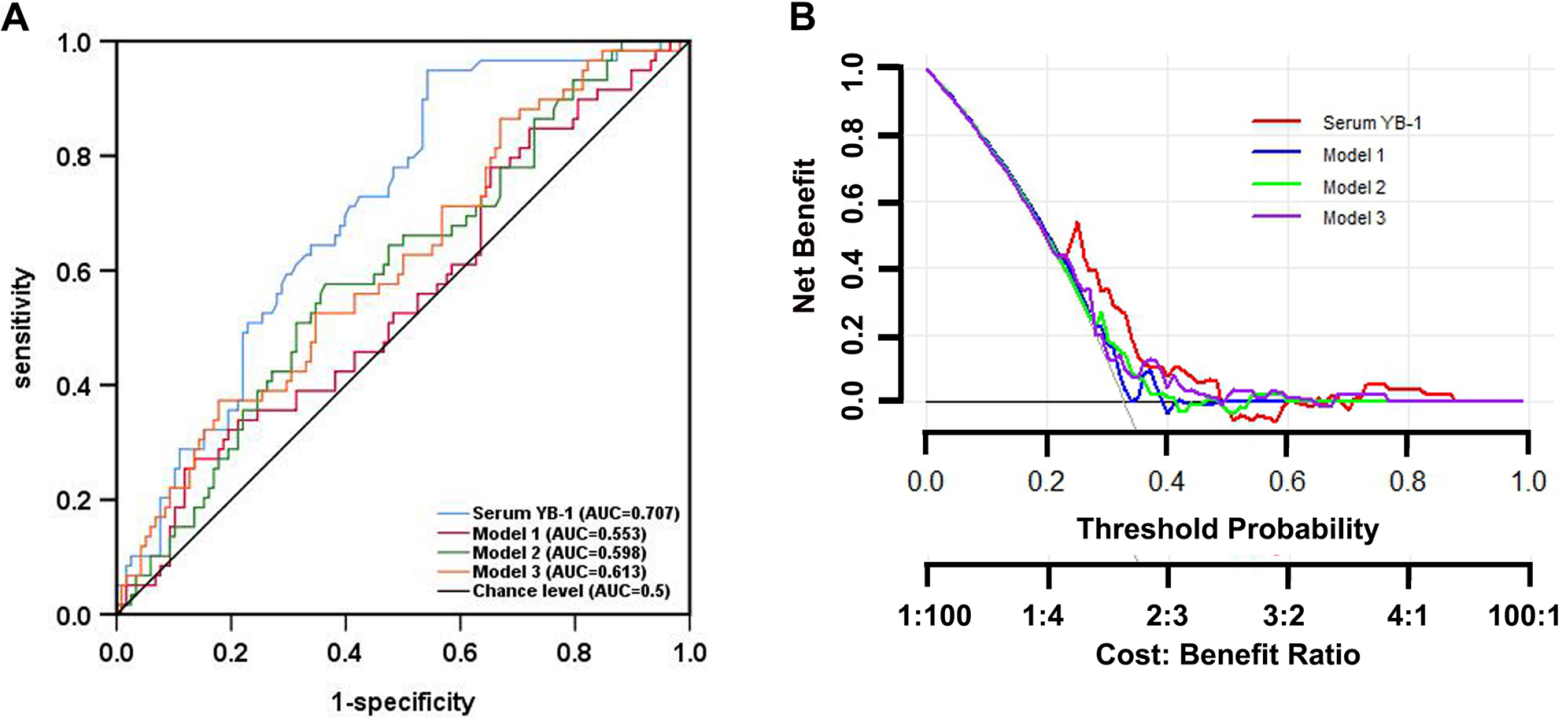
Predictive performance and decision curve analysis of serum YB-1 and multivariable models for vascular calcification. **A** ROC curves comparing serum YB-1 (blue, AUC = 0.707) with traditional models (Model 1: AUC = 0.553; Model 2: AUC = 0.598; Model 3: AUC = 0.613). **B** The red line indicates net benefit of YB-1 alone, blue indicated Model 1 (bone-mineral), green indicates Model 2 (glucose), and purple indicates Model 3 (lipid). DCA demonstrating that serum YB-1 provided higher net clinical benefit in the threshold range of 0.24–0.33

During the 30-month follow-up period, 60 patients (28.7%) developed new VC, with a higher incidence in HLP than in CON group (37.1% vs. 21%; *P* = 0.012) (Table 1). Baseline comparisons between patients with and without VC revealed that VC was strongly associated with calcium-phosphorus levels, lipid and glucose metabolism (Supplementary Table 2). ROC analysis showed that serum YB-1 alone had superior predictive value for VC (AUC 0.707, 95% CI 0.630–0.784) compared with traditional models based on bone metabolism (AUC 0.553), lipid parameters (AUC 0.613), or glucose metabolism (AUC 0.598) (Fig. 3a). Adding serum YB-1 improved overall model performance (Table 4). In the DCA plot, incorporating YB-1 yielded the highest net clinical benefit in the threshold range of 0.24–0.33 (Fig. 3b), which corresponded approximately to the intermediate predicted-risk zone for VC. Notably, serum YB-1 levels showed no significant baseline correlation with serum phosphate (*r* = 0.105, *P* = 0.514; Fig. 2c) or calcium levels (*r* = 0.105, *P* = 0.076; Fig. 2d).

**Table 4:** Performance of YB-1 and/or clinical data for predicting vascular calcification in HD patients

	AUC (95% CI) (*n* = 209)
Univariable model of biomarker	
Serum YB-1	0.707 (0.630–0.784)
Clinical models	
Model 1 (containing phosphorus, calcium, iPTH and vitamin D)	0.553 (0.463–0.643)
Model 2 (containing diabetes, glucose and HbA1c)	0.598 (0.511–0.685)
Model 3 (containing cholesterol, triglyceride, LDL-C, HDL-C and ApoB)	0.613 (0.526-0.700)
Models of clinical data and biomarker	
Serum YB-1 + Model 1	0.687 (0.603–0.770)
Serum YB-1 + Model 2	0.704 (0.628–0.781)
Serum YB-1 + Model 3	0.695 (0.613–0.777)
Serum YB-1 + Model 1 + Model 2 + Model 3	0.711 (0.633–0.790)

*YB-1* Y-box binding protein-1

### NET activation is enhanced in hyperlipidemic MHD patients

**Fig. 4:**
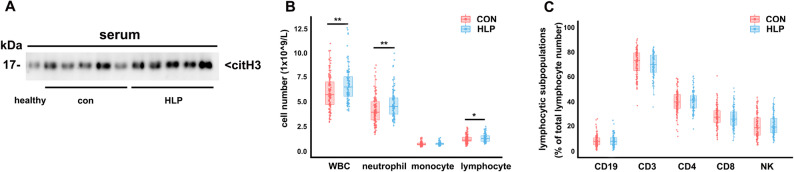
Increased NET formation and neutrophil counts in hyperlipidemia MHD patients. **A** Serum citrullinated histone H3 (citH3) levels in control (Con) and hyperlipidemia (HLP) groups. **B** Absolute counts of circulating immune cell subtypes; (**C**) Relative distribution of lymphocyte subsets (CD3+, CD4+, CD8+, CD19+, and NK cells); group means ± SD are plotted; individual values are overlaid as open circles. *P < 0.05; **P < 0.01

Given the established role of neutrophils in cardiovascular pathology, systemic NET formation was examined. Serum levels of citH3, a specific marker of NETs, were markedly higher in patients with hyperlipidemia, indicating enhanced neutrophil activation in these patients (Fig. 4a). Consistent with this, hyperlipidemic patients also exhibited significantly higher circulating neutrophil counts (4.6 ± 1.5 vs. 4.1 ± 1.5, respectively; *P* = 0.004) (Fig. 4b), while lymphocyte subsets proportions showed no significant differences between groups (Fig. 4c). Moreover, serum lipid levels did not significantly affect cytokine or chemokine levels (Supplementary Table 3).

### Extracellular YB-1 promotes neutrophil lipid accumulation and NET formation

**Fig. 5:**
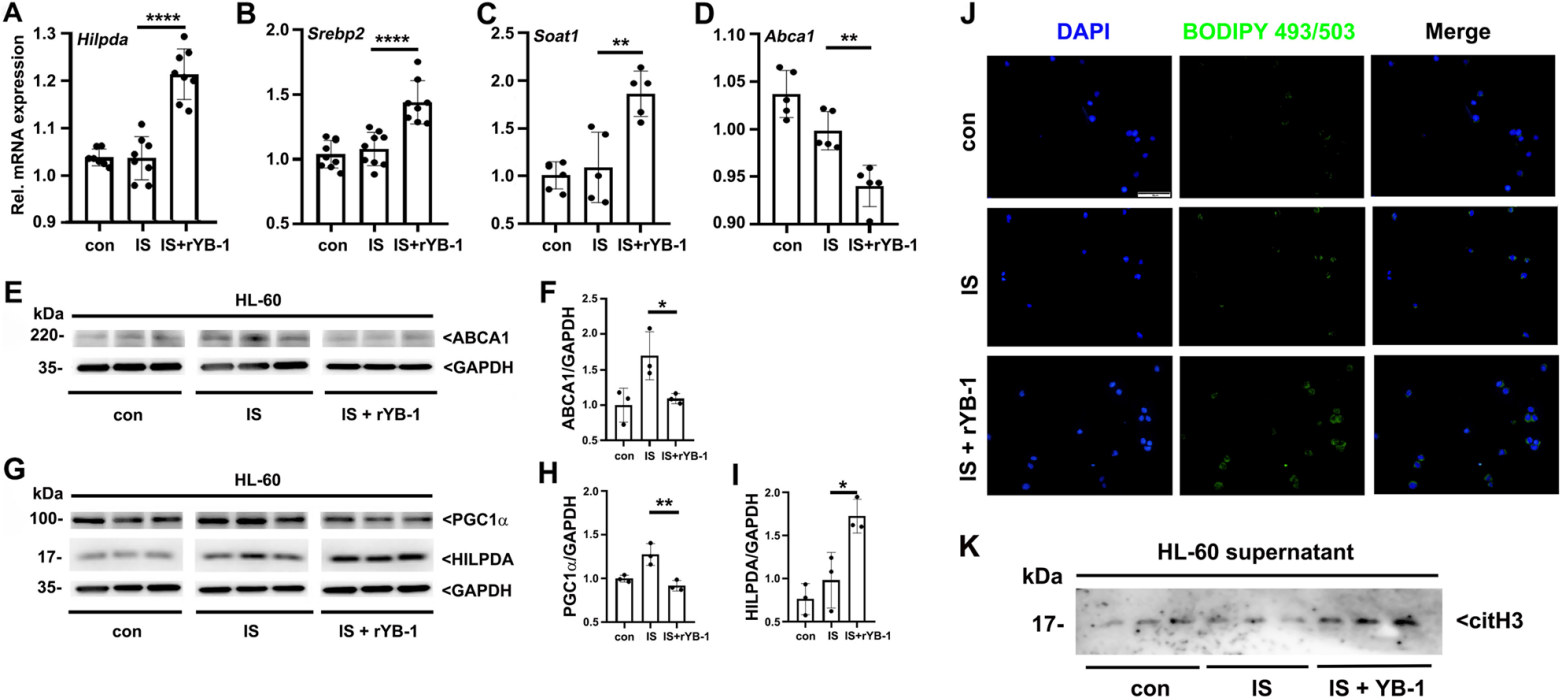
YB-1 and indoxyl sulfate promote lipid accumulation and NET formation in neutrophil-like HL-60 cells. **A**-**D** Relative mRNA expression of lipid metabolism-related genes: Hilpda, Srebp2, Soat1 (lipid synthesis), and ABCA1 (lipid efflux) in HL-60 cells treated with IS (1mM) and rYB-1 (100ng/ml) for 3 h after RA-induced differentiation (*n* = 5–9). **E-I** Western blot analysis and quantification of ABCA1, PGC1a, and Hilpda proteins, normalized to GAPDH. **J** Immunofluorescence staining showing lipid droplet accumulation (BODIPY 493/503, green) and nuclei (DAPI, blue); Scale bars, 50 mm. **K** Western blot detection of citH3 in cell culture supernatants demonstrating increased extracellular NET-associated citH3 release upon co-stimulation with IS and rYB-1. *P < 0.05; **P < 0.01; ****P < 0.0001. YB-1, Y-box binding protein-1; HL-60, human promyelocytic leukemia; IS, indoxyl sulfate; rYB-1, recombinant Y-box binding protein-1

The effect of extracellular YB-1 on neutrophil activation was first assessed to investigate its potential mechanistic link with vascular injury in a uremic model. Differentiated HL-60 cells were stimulated with the uremic toxin IS and rYB-1. Co-stimulation with IS and rYB-1 significantly increased the expression of lipid synthesis genes (Hilpda, Srebp2, and Soat1), and suppressed lipid efflux and fatty acid oxidation regulation (ABCA1 and PGC1a), suggesting enhanced intracellular lipid accumulation (Fig. 5a-i). Immunofluorescence staining confirmed increased lipid droplet accumulation in neutrophil-like cells exposed to rYB-1 (Fig. 5j).

In parallel, culture supernatants from IS and rYB-1 treated HL-60 cells were analyzed for citH3. The combination of IS and rYB-1 resulted in a marked increase in citH3 release, supporting a synergistic role of extracellular YB-1 and uremic toxins in inducing NETs (Fig. 5k).

### YB-1-induced NETs mediate calcification in vascular smooth muscle cells

**Fig. 6:**
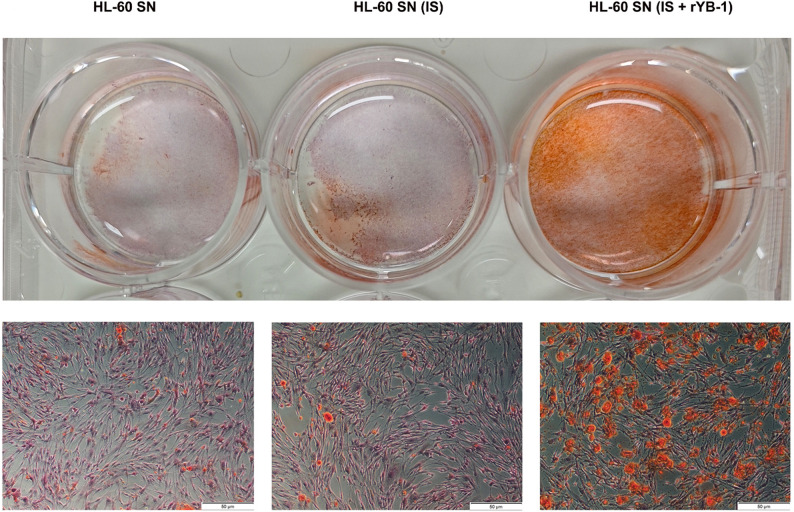
NET-rich supernatants promote vascular smooth muscle cell calcification. Human VSMCs were incubated for 72 h in osteogenic medium supplemented with culture supernatants from RA-differentiated HL-60 cells pretreated with IS (1 mM) and rYB-1 (100 ng/ml). Calcium deposition was visualized using Alizarin Red S staining. Scale bars, 50 mm

The direct effect of NETs generated under uremic and inflammatory conditions on VSMCs was then evaluated to assess their contribution to vascular injury. NET-rich supernatants from HL-60 cells stimulated with IS and rYB-1, which showed pronounced NET formation, were incubated with human VSMCs in osteogenic medium. Alizarin red staining revealed significantly increased calcium deposition in VSMCs exposed to NETs-containing supernatants, especially from both IS and rYB-1 group, compared to controls (Fig. 6). These results indicate that NETs formed under the influence of YB-1 and uremic toxins possess direct pro-calcific activity on VSMCs, offering a functional link between neutrophil activation and VC.

### IS aggravates YB-1 release, NETs activation, and vascular calcification in a CKD rat model

**Fig. 7:**
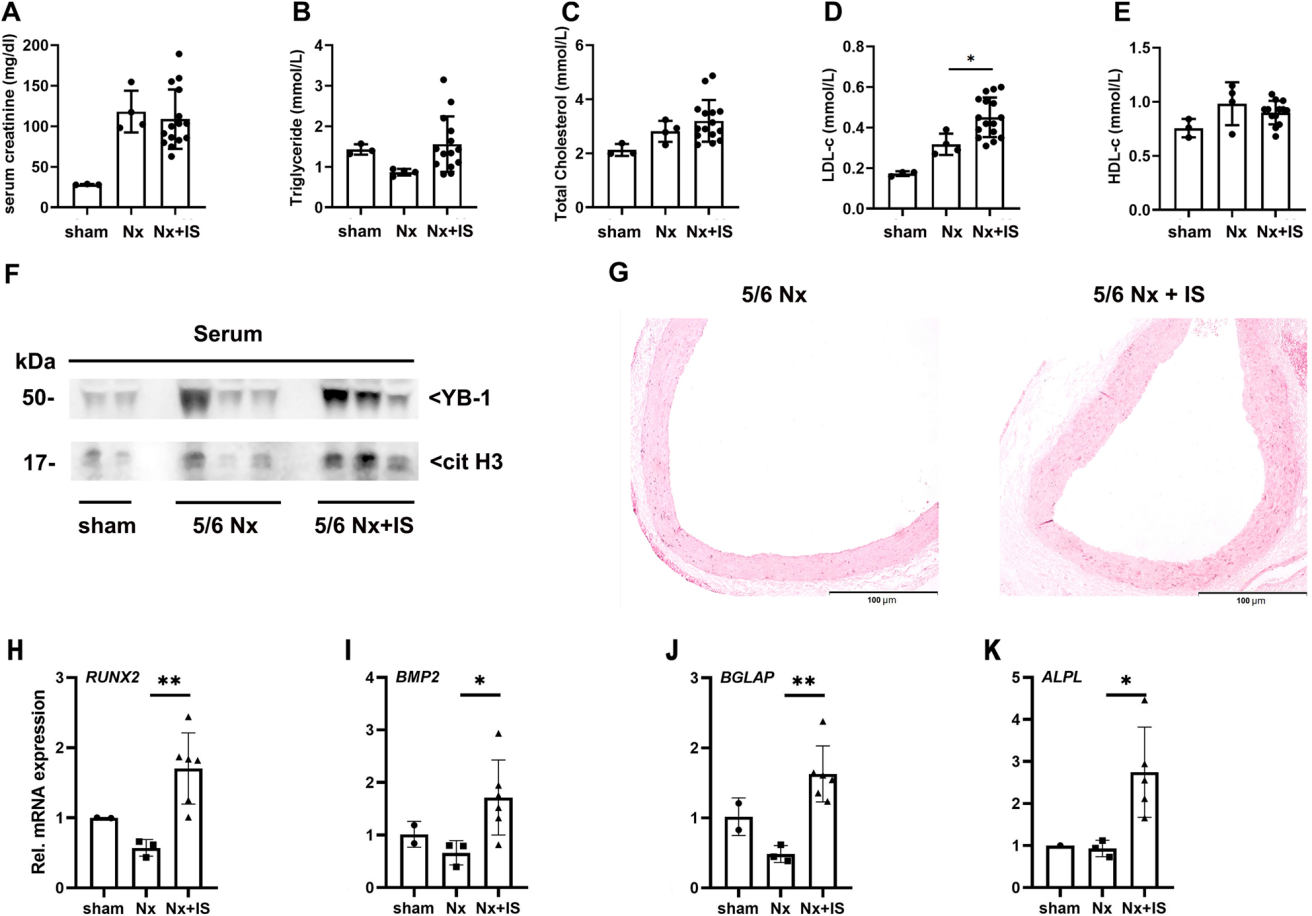
Effects of indoxyl sulfate on lipid profile, YB-1 expression, NET formation, and vascular calcification in 5/6 nephrectomized rats. Serum creatinine (**A**), serum lipid parameters (**B**-**E**), serum YB-1 and citH3 levels (**F**) in 5/6 Nx rats treated with saline or IS for 14 weeks. (**G**) Representative images of Alizarin Red S staining in aortas showing VC. Scale bars, 100 mm. (**H**-**K**) Relative mRNA expression of osteogenic differentiation genes: RUNX2, BMP2, BGLAP and ALPL. *P < 0.05; **P < 0.01; ****P < 0.0001. IS, indoxyl sulfate; 5/6 Nx, 5/6 nephrectomy; citH3, citrullinated histone H3; YB-1, Y-box binding protein-1

To validate these findings in vivo, a 5/6 nephrectomy (5/6 Nx) rat model was employed and IS was administered to stimulate uremia. After 14 weeks, IS-treated rats displayed significantly higher serum LDL-C levels than 5/6 Nx rats, whereas HDL-C, total cholesterol, triglycerides, and serum creatinine remained comparable between groups (Fig. 7a-e). Notably, serum YB-1 and citH3 levels were both significantly elevated in the IS group (Fig. 7f), confirming systemic NET activation under uremic conditions. Alizarin red staining of aortas revealed markedly increased VC in the IS group compared with 5/6 Nx rats (Fig. 7g). Co-stimulation with 5/6 Nx and IS significantly increased the expression of osteogenic differentiation genes (RUNX2, BMP2, BGLAP and ALPL)(Fig. 7h-k). These in vivo findings corroborate the clinical and cellular evidence, suggesting that IS-induced YB-1 expression promotes neutrophil-driven vascular injury in CKD.

Collectively, these clinical, cellular, and animal data strongly support a mechanistic axis in which uremic toxins induce YB-1 expression, leading to lipid accumulation in neutrophils, NETs activation, and VC, especially in the context of dyslipidemia in MHD patients.

## Discussion

This study identifies serum YB-1 as a novel biomarker for VC in MHD patients and mechanistically links dyslipidemia to NET-mediated vascular injury. In vitro and in vivo, extracellular YB-1, particularly under uremic conditions, reprogrammed neutrophil lipid metabolism, enhanced NET formation, and subsequently promoted VSMC calcification, hereby establishing a mechanistic connection between dyslipidemia to vascular injury. Clinically, YB-1 demonstrated superior predictive performance for VC compared with traditional lipid-, glucose-, and bone-metabolism models and yielded tangible net clinical benefit on decision curve analysis.

Mechanistically, prior studies have outlined the multiple roles that YB-1 plays in inflammatory processes and the regulation of lipid metabolism. YB-1 modulates fatty acid synthesis via SREBP pathways [12] and regulates cholesterol uptake in macrophages under oxidized LDL conditions [16]. However, its role in chronic kidney disease, particularly in the MHD population, remained largely unexplored. This study expands on earlier findings in ApoE-deficient mice [17] by confirming that YB-1 is not only associated with dyslipidemia but may also actively participate in lipid metabolic dysregulation under uremic conditions. These data collectively support a mechanistic link between YB-1 dysfunction and lipid disorder, providing a biological basis for its observed elevation in hyperlipidemic MHD patients. This bridging evidence connects basic lipid regulatory mechanisms to clinical metabolic abnormalities, suggesting that YB-1 serves as a molecular connector between dyslipidemia and vascular injury.

Importantly, this study report for the first time that YB-1 exhibits a strong correlation with NET formation in hyperlipidemic MHD patients, as evidenced by elevated serum citH3 levels. This aligns with findings that hyperlipidemia promotes NETosis in atherosclerosis [18]. In vitro studies show that co-treatment with IS and rYB-1 markedly increased NET release and lipid synthesis gene expression in HL-60-derived neutrophil-like cells, indicating that uremic and inflammatory stimuli act synergistically to drive neutrophil activation. These findings build upon previous observations that IS alone primes NETosis in CKD [19] and that extracellular YB-1 is a structural component of NETs [13].

Notably, this study also extends the biological significance of NETs by demonstrating their direct pro-calcific effects on VSMCs. This supports the concept that neutrophil-derived NETs actively contribute to the pathogenesis of VC [8, 20]. Moreover, in vivo 5/6 nephrectomy rat model treated with IS showed increased serum LDL-C, YB-1, and citH3 levels, along with pronounced aortic calcification. Although the IS rat model does not fully replicate the dialysis environment, it reinforces the mechanistic relevance of YB-1-mediated NET activation and its systemic impact on vascular pathology in uremic conditions. These findings integrate molecular and experimental evidence to establish a YB-1-NET-VSMC axis that mechanistically links dyslipidemia, NET activation and VC. This mechanistic insight provides a foundation for assessing the clinical relevance of YB-1 in predicting VC risk among MHD patients.

Clinically, these mechanistic insights translate into measurable differences in serum YB-1 levels among MHD patients, providing a rationale for its application as a biomarker for VC risk. Conventional lipid markers often performed poorly in this setting due to “reverse epidemiology”, where lower cholesterol levels paradoxically associate with worse outcomes [3]. YB-1, integrating inflammatory and metabolic cues, demonstrated superior predictive accuracy for VC compared with traditional lipid-, glucose-, and bone-metabolism models. While ROC analysis reflects the discriminative ability of a single biomarker, DCA provided complementary evidence that YB-1 offers greater net clinical benefit within the 0.24–0.33 threshold range, corresponding to patients at intermediate predicted VC risk. This suggests that YB-1 may help refine individualized risk stratification and guide preventive interventions in MHD populations.

### Strengths and limitations

This investigation is the first to uncover serum YB-1 as a novel biomarker for VC in MHD patients and uncover its mechanistic role in linking dyslipidemia to NET-mediated vascular injury. Extracellular YB-1 under uremic conditions reprograms neutrophil lipid metabolism, promotes NET formation, and drives VSMC calcification. Clinically, YB-1 outperforms traditional metabolic markers, offering a novel, mechanistically informed tool for personalized VC risk stratification. Overall, these findings offer positive potential for the clinical transformation of YB-1.

However, this study has several limitations. First, VC was assessed only in coronary arteries and thoracic aorta, excluding abdominal vasculature. Second, the measurement of serum YB-1 and NETs markers at a single time point limited assessment of temporal dynamics. Third, mechanistic experiments primarily employed a gain-of-function strategy using rYB-1 without genetic silencing or neutralization. Although this approach reflects the clinical relevance of circulating YB-1, future studies using loss-of-function techniques are warranted to validate its causal role in NET formation and VC progression. Finally, although the sample size was sufficient for initial discovery, it constrains the scope of subgroup analyses and limits the generalizability of the findings.

## Conclusion

This study identifies YB-1 as a promising biomarker for VC in MHD patients. It provides incremental discriminatory power beyond traditional lipid, glucose, and bone markers, and offers measurable clinical net benefit within intermediate-risk decision thresholds. Mechanistically, extracellular YB-1 enhances neutrophil lipid accumulation and NET release under uremic stress, promoting VSMCs calcification. These observations point to the potential relevance of the YB-1-NET axis in refining risk stratification and guiding therapeutic strategies, aimed at reducing cardiovascular complications and improving long-term health outcomes in MHD population.

## Supplementary Information

Supplementary Material 1.

Supplementary Material 2.

Supplementary Material 3.

## Data Availability

The datasets generated and analyzed during the current study are available from the corresponding authors upon reasonable request.
