# Supplementary material for: Serum YB-1 links dyslipidemia to NET-mediated vascular calcification in hemodialysis
Source: Lipids Health Dis. 2026 Jan 13;25:46. doi: 10.1186/s12944-025-02832-y (PMC12888723; doi:10.1186/s12944-025-02832-y)
Supplement: Supplementary file 1 — Supplementary Material 1. [file 12944_2025_2832_MOESM1_ESM.docx]

**Materials**

**Protein extraction and Western blot analysis**

Protein extraction and Western blotting were performed as previously described ^[13]^. Primary antibodies used included HILPDA (1:1000; Antibodies-online, PA, USA), PGC1α (1:1000; Abcam, MA, USA), ABCA1 (1:1000; Novus Biologicals, Colorado, USA), citH3 (1:1000; Abcam, MA, USA) and YB-1 (1:1000; Sigma-Aldrich, St Louis, MO, USA). Equal protein loading was verified by probing the blots with a monoclonal anti-GAPDH antibody (Santa Cruz Biotechnology, TX, USA).

**Quantitative real-time PCR**

Total RNA was extracted from the cells using TRIzol reagent (Sigma-Aldrich) according to the manufacturer’s instructions. cDNA was synthesized using a PrimeScript RT Master Mix Kit (Takara, Tokyo, Japan). Quantitative real-time PCR (qRT-PCR) was performed using TaqMan probes (Applied Biosystems) or primers with the SYBR Green Dye Detection System (Supplemental Table 1), as described previously. Gene expression levels were calculated using the comparative ΔCT method.

**Tables**

**Table 1. Gene primers**

|  | Forward Primer sequence | Reverse Primer sequence |
| --- | --- | --- |
| 18S | 5’-CGGCTACCACATCCAAGGAA-3’ | 5’-CCTGTATTGTTATTTTTCGTCACTACCT-3’ |
| Hilpda | 5’-AAGCATGTGTTGAACCTCTACC-3’ | 5’-TGTGTTGGCTAGTTGGCTTCT-3’ |
| Srebp2 | 5’-CTGCAACAACAGACGGTAATGA-3’ | 5’-CCATTGGCCGTTTGTGTCAG-3’ |
| Soat1 | 5’-CAAGGCGCTCTCTCTTAGATG-3’ | 5’-GGTCCAAACAACGGTAGGAAA-3’ |
| Abca1 | 5’-ACCCACCCTATGAACAACATGA-3’ | 5’-GAG TCGGGTAACGGAAACAGG-3’ |
| Runx2 | 5’- GACTGTGGTTACCGTCATGGC -3’ | 5’- ACTTGGTTTTTCATAACAGCGGA -3’ |
| Bmp2 | 5’- GCAGGTCTTTGCACCAAGATG -3’ | 5’- TTTTCCGTCGTGGCCAAAAG -3’ |
| Bglap | 5’- TGAGCTCAACCCCAATTGTG -3’ | 5’- AAACGGTGGTGCCATAGATG -3’ |
| Alpl | 5’- ATGGATGAGGCCATCGGAAAG -3’ | 5’- AGCCACCAAACGTGAAAACG -3’ |

**Table 2**. **Clinical baseline characteristics of HD patients with and without vascular calcification**

| **Variables** | **Overall**  **(n=209)** | **CON**  **(n=71)** | **Calcification (n=138)** | ***P* value** |
| --- | --- | --- | --- | --- |
| Age, y | 59.7 ± 15.2 | 50.7 ± 14.0 | 64.4 ± 13.6 | <0.001*** |
| Male, N (%) | 134 (64.1%) | 49.0 (69.0%) | 85.0 (61.6%) | 0.290 |
| Diabetes, N (%) | 47 (22.5%) | 6.0 (8.5%) | 41.0 (29.7%) | <0.001*** |
| Hypertension, N (%) | 184 (88.0%) | 57.0 (80.3%) | 127.0 (92.0%) | 0.013* |
| Dialysis vintage, y | 6.1 ± 4.7 | 5.4 ± 4.2 | 6.4 ± 4.8 | 0.117 |
| spKt/V | 1.3 ± 0.5 | 1.2 ± 0.2 | 1.3 ± 0.6 | 0.449 |
| C reactive protein, mg/L | 4.0 (1.2, 9.3) | 3.7 (1.3, 10.6) | 3.7 (1.3, 10.4) | 0.286 |
| Anemia |  |  |  |  |
| Hemoglobin, g/L | 117.0 ± 17.5 | 116.3 ± 17.0 | 117.3 ± 17.9 | 0.690 |
| Ferritin, ng/ml | 177.0 (103.5, 352.5) | 176.0 (104.5, 357.5) | 178.0 (106.0, 354.0) | 0.201 |
| Transferrin saturation, % | 26.0 (18.0, 38.0) | 26.0 (18.5, 37.0) | 26.0 (19.0, 38.0) | 0.522 |
| Albumin, g/L | 39.3 ± 3.5 | 40.0 ± 3.6 | 38.9 ± 3.4 | 0.037* |
| Glucose metabolism |  |  |  |  |
| Glucose, mmol/L | 7.4 ± 3.3 | 6.6 ± 2.9 | 7.8 ± 3.5 | 0.013* |
| HbA1c, % | 5.8 ± 1.0 | 5.5 ± 0.8 | 5.9 ± 1.1 | 0.004** |
| Lipid metabolism |  |  |  |  |
| Cholesterol, mmol/L | 3.9 ± 1.0 | 3.7 ± 1.0 | 4.0 ± 1.0 | 0.095 |
| Triglyceride, mmol/L | 1.9 ± 1.2 | 1.9 ± 1.3 | 1.9 ± 1.1 | 0.658 |
| LDL-c, mmol/L | 2.0 ± 0.8 | 1.9 ± 0.8 | 2.1 ± 0.8 | 0.036* |
| HDL-c, mmol/L | 1.1 ± 0.4 | 1.1 ± 0.4 | 1.1 ± 0.4 | 0.751 |
| ApoB, g/L | 0.7 ± 0.2 | 0.6 ± 0.2 | 0.7 ± 0.2 | 0.065 |
| Bone metabolism |  |  |  |  |
| Calcium, mmol/L | 2.3 ± 0.2 | 2.4 ± 0.2 | 2.3 ± 0.2 | 0.335 |
| Phosphate, mmol/L | 2.3 ± 0.7 | 2.5 ± 0.7 | 2.2 ± 0.6 | 0.011* |
| iPTH, pg/ml | 309.1 ± 209.1 | 315.7 ± 228.0 | 305.8 ± 199.8 | 0.748 |
| 25(OH)D, nmol/L | 32.3 ± 14.5 | 32.2 ± 13.5 | 32.3 ± 15.0 | 0.944 |
| cTnT, ng/ml | 0.1 ± 0.1 | 0.1 ± 0.1 | 0.1 ± 0.1 | 0.002** |

The data in the table are expressed as mean ± standard deviation or number (%); **P*<0.05; ***P*<0.01; ****P*<0.001.

**Table 3**. Chemokines/cytokines in HD patients at baseline

| **Variables** | **Overall (n=209)** | **CON (n=112)** | **HLP (n=97)** | ***P* value** |
| --- | --- | --- | --- | --- |
| IL-1β, pg/ml | 5.1 (5.0, 8.1) | 5.1 (5.0, 8.1) | 5.1 (5.0, 8.1) | 0.616 |
| IL-2R, U/ml | 1329.3 ± 557.0 | 1359.2 ± 472.6 | 1295.3 ± 640.4 | 0.414 |
| IL-6, pg/mL | 4.6 (3.1, 8.0) | 4.6 (3.1, 7.8) | 4.6 (3.1, 7.7) | 0.998 |
| IL-8, pg/mL | 15.0 (9.0, 38.5) | 15.0 (9.0, 38.5) | 15.0 (9.0, 38.0) | 0.666 |
| TNF, pg/mL | 18.3 ± 6.5 | 18.5 ± 6.0 | 18.1 ± 7.2 | 0.704 |

The data in the table are expressed as mean ± standard deviation.
