# Supplementary material for: Serum YB-1 links dyslipidemia to NET-mediated vascular calcification in hemodialysis
Source: Lipids Health Dis. 2026 Jan 13;25:46. doi: 10.1186/s12944-025-02832-y (PMC12888723; doi:10.1186/s12944-025-02832-y)
Supplement: Supplementary file 3 — Supplementary Material 3. [file 12944_2025_2832_MOESM3_ESM.pdf]

Serum YB-1 links dyslipidemia to NET-mediated vascular calcification in hemodialysis

By jiaxin chen

WORD COUNT	5022	TIME SUBMITTED	04-DEC-2025 08:46AM
		PAPER ID	119309860

Serum YB-1 links dyslipidemia to NET-mediated vascular calcification in hemodialysis

Formatted: Shadow

Jiaxin Chen[1#], Li Wang[1#], Shuan Zhao[1], Jian Zhang[1], Nana Song[1], Yi Fang[1], Zhen Zhang[1], Xuesen Cao[1], Bo Shen[1], Jie Teng[1,2], Jianzhou Zou[1], Jieru Cai[1], Xiaoqiang Ding[1*], Jialin Wang[1*]

Formatted: Shadow

Abstract

Background Vascular calcification (VC) is highly prevalent in patients undergoing maintenance hemodialysis (MHD) and is associated with cardiovascular morbidity. However, traditional lipid and mineral markers have limited predictive value. Y-box binding protein-1 (YB-1), a regulator of lipid metabolism and inflammation, may provide additional mechanistic and clinical insight.

Methods Serum YB-1 was measured in 209 MHD patients (30-month follow-up) who were stratified into hyperlipidemia and control groups. Receiver Operating Characteristic (ROC) and decision curve analysis (DCA, threshold range 0.1-0.4) were used to assess the predictive performance of YB-1 against traditional models based on lipid, glucose, and bone metabolism. Mechanistic studies in HL-60-derived neutrophil-like cells and a 5/6 nephrectomized rat model were performed to assess the role of YB-1 in neutrophil extracellular trap (NET) formation and VC.

Results Serum YB-1 was significantly elevated in hyperlipidemia patients and was independently associated with new-onset VC (AUC 0.707, 95% CI 0.630-0.784). YB-1 outperformed lipid-, glucose-, and bone-based models, providing added net clinical benefit in DCA within the 0.24-0.33 threshold range. Mechanistically, serum levels of citrullinated histone H3 (citH3), a NET marker, were increased in hyperlipidemia patients. In vitro, YB-1 and IS synergistically enhanced neutrophil lipid droplet accumulation and citH3 release, while NET-rich supernatants promoted VSMC

Formatted: Shadow

calcification. In vivo, IS-treated 5/6 nephrectomy rats displayed elevated YB-1, increased citH3, and aggravated aortic calcification.

Conclusion Serum YB-1 is a novel predictor and potential mechanistic mediator of VC in MHD patients. Incorporating YB-1 into existing clinical risk models may support earlier recognition of individuals at elevated cardiovascular risk and inform more effective management strategies to improve long-term health outcomes.

Keywords: Y-Box-Binding Protein 1, vascular calcification, hemodialysis, neutrophil extracellular traps, dyslipidemias

Formatted: Shadow

Highlights

1. Extracellular YB-1 reprograms neutrophil lipid metabolism to enhance NET formation and drive VSMC calcification under uremic conditions.
2. Clinically, serum YB-1 levels are independently associated with dyslipidemia in MHD patients.
3. YB-1 acts as a mechanistic bridge linking dyslipidemia to vascular calcification in MHD patients.
4. Integrating YB-1 improves vascular calcification prediction beyond traditional metabolic markers, providing measurable net clinical benefit in intermediate-risk MHD patients.

Formatted: Shadow

Introduction

Vascular calcification (VC) is a key contributor to incidence of cardiovascular events and fatal outcomes in end-stage renal disease (ESRD) patients, particularly among patients undergoing maintenance hemodialysis (MHD) (1). Although dyslipidemia contributes significantly to the risk of cardiovascular risk in the general population, its association with outcomes in dialysis patients is paradoxical (“reverse epidemiology”) (2–3), and statin trials have largely failed to demonstrate significant cardiovascular benefits in this setting (4–5). These observations question the adequacy of traditional lipid markers for risk stratification in MHD.

Beyond lipid abnormalities, uremic toxins, chronic inflammation, and immune dysregulation shape the cardiovascular landscape in ESRD (6–7). Neutrophil extracellular traps (NETs) have been recognized as critical drivers of vascular injury and calcification (8–9). Y-box binding protein-1 (YB-1) is a multifunctional cold shock protein implicated in lipid metabolism and inflammatory signaling (10–12) and has been recognized as a constituent of NETs (13). Whether YB-1 functionally links dyslipidemia to NET-driven vascular injury and whether it improves VC risk prediction in MHD remain unclear.

The aim of this study was to investigate the clinical relevance and functional role of YB-1 in patients undergoing MHD, hypothesizing that it functions not merely as a circulating biomarker but also as an active mediator that promotes vascular injury

Formatted: Shadow

through neutrophil lipid reprogramming and NET formation, ultimately driving VC. To test this, a retrospective clinical cohort was combined with mechanistic *in vitro* experiments and an *in vivo* 5/6 nephrectomy rat model. This integrated approach extends previous descriptive findings toward establishing a causal and translational link between YB-1 activation, dyslipidemia, and VC in MHD patients.

Methods

Cohort Description and Study Design

This cohort study enrolled adult patients (>18 years) undergoing MHD at Zhongshan Hospital, Fudan University, between November 2022 and March 2023. Inclusion criteria required patients to be on regular dialysis for at least 3 months. Exclusion criteria included recent infection or myocardial infarction (<1 month), use of lipid-lowering medications, inability to provide informed consent, or missing serum samples. A total of 209 eligible patients were included (Figure 1). Patients were stratified into a hyperlipidemia (HLP) group (total cholesterol >5.2 mmol/L and/or triglycerides >1.7 mmol/L) and a control (CON) group in accordance with the 2016 Chinese Guidelines for the Management of Dyslipidemia in Adults (14). Baseline demographics, comorbidities, dialysis vintage, and laboratory parameters (including full lipid panel, glucose/HbA1c, and mineral metabolism indices) were compared between HLP and CON groups. Variables that showed potential imbalance ($P < 0.05$) were

Formatted: Shadow

subsequently adjusted for in multivariable analyses to minimize confounding. Because of the limited sample size, formal propensity matching was not performed; instead, adjusted regression models were used to ensure comparability between groups. VC was assessed by coronary computed tomography angiography and carotid ultrasound, and incident VC events were prospectively recorded during a 30-month follow-up period. This study was approved by the Institutional Review Board of Zhongshan Hospital (approval number: B2021-740) and conducted in accordance with the Declaration of Helsinki, with all participants providing written informed consent.

Laboratory measurement

Blood samples were obtained pre-dialysis from arterial vascular access. Serum was separated by centrifugation and stored at -80°C. YB-1 levels were quantified by a commercially available ELISA kit (LifeSpan BioSciences, WA, USA) following the manufacturer's instructions. High-sensitivity C-reactive protein (hs-CRP), lipid levels (total cholesterol, triglycerides, LDL-C, HDL-c), creatinine, phosphate, calcium, and complete blood counts were assessed by means of standardized clinical laboratory protocols in the Department of Laboratory Medicine at Zhongshan Hospital.

Peripheral lymphocyte subsets (CD3+, CD4+, CD8+, CD19+, and NK cells) were analyzed using flow cytometry (BD Biosciences, USA) (15). Serum levels of cytokines (tumor necrosis factor (TNF)-a, interleukin (IL)-1b, IL-2R, IL-6, and IL-8) were quantified by ELISA (R&D systems, USA) according to the manufacturer's protocols.

Formatted: Shadow

All samples were analyzed in duplicate.

In vitro neutrophil stimulation and lipid metabolism assay

HL-60 promyelocytic leukemia cells (ATCC, USA) were cultured in RPMI 1640 with 10% FBS. Neutrophil differentiation was induced using 1mM retinoic acid (RA; Sigma-Aldrich, St Louis, MO, USA) for 72 h. Differentiated cells were treated with 1 mM indoxyl sulfate (IS) (Sigma-Aldrich) and 100 ng/ml recombinant YB-1 (rYB-1; Abnova, Taiwan, China) for another 3 hours.

Lipid metabolism-related genes (Hilpda, Srebp2, Soat1, ABCA1, and PGC1a) was analyzed by qRT-PCR. Protein levels (YB-1 and citrullinated histone H3(citH3)) were assessed by Western blot. Lipid droplets were visualized with BODIPY 493/503 staining (Beyotime Biotechnology, Shanghai, China) according to the manufacturer's instructions.

NETs-VSMC calcification assay

To assess the functional consequences of neutrophil activation, cell culture supernatants were collected from HL-60 cells treated with IS and rYB-1. These NETs-rich supernatants were combined with vascular smooth muscle cells (VSMCs) culture medium at a 2:1 ratio and co-incubated with human VSMCs for 72h in osteogenic medium. VSMC calcification was assessed using Alizarin Red S staining.

Animal model of uremia and vascular calcification

Male Sprague-Dawley rats (180-220g) were purchased from SLAC Laboratory

Formatted: Shadow

animal Co. LTD, Shanghai, China. The 5/6 nephrectomy (5/6 Nx) operation was performed to induce chronic kidney disease (CKD). Four weeks after surgery, rats were randomly assigned to receive IS (100mg/kg, twice a week, intraperitoneally) or saline alone as control for 14 weeks.

At endpoint, serum samples were analyzed for YB-1, lipid profiles, creatinine, and citH3. Aortas were harvested and subjected to Alizarin Red staining to evaluate VC. VC-related genes (RUNX2, BMP2, BGLAP, and ALPL) was analyzed by qRT-PCR. Experimental rats were humanely sacrificed by decapitation. All experimental protocols received approval from the Institutional Animal Care and Use Committee of Fudan University (Approval No. 2023-109) and adhered strictly to the National Institutes of Health Guide for laboratory animal care. Measures have been implemented throughout this study to reduce animal suffering to the greatest extent possible.

Statistical analysis

Continuous variables were expressed as mean ± standard deviation (SD) or median (interquartile range, IQR) as appropriate, and categorical variables as counts and percentages. Statistical comparisons between the HLP and CON groups were performed using either Student' s t-test for continuous variables or the chi-square test for categorical variables, depending on the type of data. The analysis of variance followed by Tukey's *post-hoc* test was used for comparisons involving more than two groups. Variables demonstrating significant differences between the two groups in the baseline

Formatted: Shadow

analysis were entered into a binary logistic regression model to determine independent predictors of hyperlipidemia. This study employed a classical maximum-likelihood binary logistic regression model as implemented in SPSS. This modelling approach does not incorporate any form of regularisation (e.g., L1 or L2). Consequently, standardisation of continuous predictors was not required. All continuous variables were retained on their original scales to preserve the interpretability and clinical relevance of the regression coefficients.

Correlations were performed to assess associations between serum YB-1 and other parameters by Spearman's rank analysis. To evaluate the predictive ability of the models, predicted probabilities for each observation were generated based on the fitted model. The model-generated probabilities served as the basis for constructing receiver operating characteristic (ROC) curves. The predictive performance of serum YB-1 for VC was evaluated separately using ROC curve analysis, with the area under the curve (AUC) compared among composite models based on lipid-, glucose-, and bone-metabolism parameters. Decision curve analysis (DCA) was additionally conducted to evaluate the net clinical benefit of YB-1 and multivariable models across a range of clinically plausible decision thresholds. All statistical analyses were performed using SPSS version 26.0 (IBM Corp., Armonk, NY, USA), GraphPad Prism 8.0 (GraphPad Software, San Diego, CA, USA) and R version 4.4.2 (R Foundation for Statistical Computing, Vienna, Austria), with two-tailed $P < 0.05$ considered statistically

Formatted: Shadow

significant. All *in vitro* experiments were conducted at least in triplicates.

Results

Baseline characteristics

Of 209 MHD patients (64.1% male; mean age 59.7 years), 97 (46.4%) were classified as HLP and 112 (53.6%) as CON (Table 1). Compared with controls, the HLP group exhibited significantly higher levels of HbA1c (6.0 ± 1.2% vs. 5.6 ± 0.8%, respectively; *P*=0.005) and phosphate (2.4 ± 0.7 vs. 2.3 ± 0.6 mmol/L, respectively; *P*=0.029), along with lower levels of 25(OH)D3 (29.0 ± 11.1 vs. 35.1 ± 16.4 nmol/L, respectively; *P*=0.002) (Tables 1 and 2). No substantial variations were observed in inflammatory and cardiovascular parameters.

Serum YB-1 concentrations were markedly elevated in the HLP group relative to the CON group (1.22 ± 0.81 vs. 0.98 ± 0.72 ng/ml, respectively; *P*=0.023). Correlation analysis indicated a positive association between serum YB-1 and both total cholesterol (r=0.201, *P*=0.004; Figure 2a) and triglyceride (r=0.192, *P*=0.005; Figure 2b). In multivariate logistic regression analysis adjusted for HbA1c, phosphate, and 25(OH)D3, serum YB-1 remained an independent predictor of dyslipidemia (*P*=0.01) (Table 3).

Serum YB-1 predicts the risk of vascular calcification in MHD patients

During the 30-month follow-up period, 60 patients (28.7%) developed new VC, with a higher incidence in HLP than in CON group (37.1% vs. 21%; *P*=0.012) (Table

Formatted: Shadow

1). Baseline comparisons between patients with and without VC revealed that VC was strongly associated with calcium-phosphorus levels, lipid and glucose metabolism (Supplementary Table 2). ROC analysis showed that serum YB-1 alone had superior predictive value for VC (AUC 0.707, 95% CI 0.630-0.784) compared with traditional models based on bone metabolism (AUC 0.553), lipid parameters (AUC 0.613), or glucose metabolism (AUC 0.598) (Figure 3a). Adding serum YB-1 improved overall model performance (Table 4). In the DCA plot, incorporating YB-1 yielded the highest net clinical benefit in the threshold range of 0.24-0.33 (Figure 3b), which corresponded approximately to the intermediate predicted-risk zone for VC. Notably, serum YB-1 levels showed no significant baseline correlation with serum phosphate (r=0.105, P=0.514; Figure 2c) or calcium levels (r=0.105, P=0.076; Figure 2d).

NET activation is enhanced in hyperlipidemic MHD patients

Given the established role of neutrophils in cardiovascular pathology, systemic NET formation was examined. Serum levels of citH3, a specific marker of NETs, were markedly higher in patients with hyperlipidemia , indicating enhanced neutrophil activation in these patients (Figure 4a). Consistent with this, hyperlipidemic patients also exhibited significantly higher circulating neutrophil counts (4.6 ± 1.5 vs. 4.1 ± 1.5, respectively; P=0.004) (Figure 4b), while lymphocyte subsets proportions showed no significant differences between groups (Figure 4c). Moreover, serum lipid levels did not significantly affect cytokine or chemokine levels (Supplementary Table 3).

Formatted: Shadow

Extracellular YB-1 promotes neutrophil lipid accumulation and NET formation

The effect of extracellular YB-1 on neutrophil activation was first assessed to investigate its potential mechanistic link with vascular injury in a uremic model. Differentiated HL-60 cells were stimulated with the uremic toxin IS and rYB-1. Co-stimulation with IS and rYB-1 significantly increased the expression of lipid synthesis genes (Hilpda, Srebp2, and Soat1), and suppressed lipid efflux and fatty acid oxidation regulation (ABCA1 and PGC1a), suggesting enhanced intracellular lipid accumulation (Figure 5a-i). Immunofluorescence staining confirmed increased lipid droplet accumulation in neutrophil-like cells exposed to rYB-1 (Figure 5j).

In parallel, culture supernatants from IS and rYB-1 treated HL-60 cells were analyzed for citH3. The combination of IS and rYB-1 resulted in a marked increase in citH3 release, supporting a synergistic role of extracellular YB-1 and uremic toxins in inducing NETs (Figure 5k).

YB-1-induced NETs mediate calcification in vascular smooth muscle cells

The direct effect of NETs generated under uremic and inflammatory conditions on VSMCs was then evaluated to assess their contribution to vascular injury. NET-rich supernatants from HL-60 cells stimulated with IS and rYB-1, which showed pronounced NET formation, were incubated with human VSMCs in osteogenic medium. Alizarin red staining revealed significantly increased calcium deposition in VSMCs exposed to NETs-containing supernatants, especially from both IS and rYB-1

Formatted: Shadow

group, compared to controls (Figure 6). These results indicate that NETs formed under the influence of YB-1 and uremic toxins possess direct pro-calcific activity on VSMCs, offering a functional link between neutrophil activation and VC.

IS aggravates YB-1 release, NETs activation, and vascular calcification in a CKD rat model

To validate these findings in vivo, a 5/6 nephrectomy (5/6 Nx) rat model was employed and IS was administered to stimulate uremia. After 14 weeks, IS-treated rats displayed significantly higher serum LDL-C levels than 5/6 Nx rats, whereas HDL-C, total cholesterol, triglycerides, and serum creatinine remained comparable between groups (Figure 7a-e). Notably, serum YB-1 and citH3 levels were both significantly elevated in the IS group (Figure 7f), confirming systemic NET activation under uremic conditions. Alizarin red staining of aortas revealed markedly increased VC in the IS group compared with 5/6 Nx rats (Figure 7g). Co-stimulation with 5/6 Nx and IS significantly increased the expression of osteogenic differentiation genes (RUNX2, BMP2, BGLAP and ALPL)(Figure 7h-k). These *in vivo* findings corroborate the clinical and cellular evidence, suggesting that IS-induced YB-1 expression promotes neutrophil-driven vascular injury in CKD.

Collectively, these clinical, cellular, and animal data strongly support a mechanistic axis in which uremic toxins induce YB-1 expression, leading to lipid accumulation in neutrophils, NETs activation, and VC, especially in the context of dyslipidemia in MHD

Formatted: Shadow

patients.

Discussion

This study identifies serum YB-1 as a novel biomarker for VC in MHD patients and mechanistically links dyslipidemia to NET-mediated vascular injury. *In vitro* and *in vivo*, extracellular YB-1, particularly under uremic conditions, reprogrammed neutrophil lipid metabolism, enhanced NET formation, and subsequently promoted VSMC calcification, hereby establishing a mechanistic connection between dyslipidemia to vascular injury. Clinically, YB-1 demonstrated superior predictive performance for VC compared with traditional lipid-, glucose-, and bone-metabolism models and yielded tangible net clinical benefit on decision curve analysis.

Mechanistically, prior studies have outlined the multiple roles that YB-1 plays in inflammatory processes and the regulation of lipid metabolism. YB-1 modulates fatty acid synthesis via SREBP pathways (12) and regulates cholesterol uptake in macrophages under oxidized LDL conditions (16). However, its role in chronic kidney disease, particularly in the MHD population, remained largely unexplored. This study expands on earlier findings in ApoE-deficient mice (17) by confirming that YB-1 is not only associated with dyslipidemia but may also actively participate in lipid metabolic dysregulation under uremic conditions. These data collectively support a mechanistic link between YB-1 dysfunction and lipid disorder, providing a biological basis for its

Formatted: Shadow

observed elevation in hyperlipidemic MHD patients. This bridging evidence connects basic lipid regulatory mechanisms to clinical metabolic abnormalities, suggesting that YB-1 serves as a molecular connector between dyslipidemia and vascular injury.

Importantly, this study report for the first time that YB-1 exhibits a strong correlation with NET formation in hyperlipidemic MHD patients, as evidenced by elevated serum citH3 levels. This aligns with findings that hyperlipidemia promotes NETosis in atherosclerosis (18). *In vitro* studies show that co-treatment with IS and rYB-1 markedly increased NET release and lipid synthesis gene expression in HL-60-derived neutrophil-like cells, indicating that uremic and inflammatory stimuli act synergistically to drive neutrophil activation. These findings build upon previous observations that IS alone primes NETosis in CKD (19) and that extracellular YB-1 is a structural component of NETs (13).

Notably, this study also extends the biological significance of NETs by demonstrating their direct pro-calcific effects on VSMCs. This supports the concept that neutrophil-derived NETs actively contribute to the pathogenesis of VC (8, 20). Moreover, *in vivo* 5/6 nephrectomy rat model treated with IS showed increased serum LDL-C, YB-1, and citH3 levels, along with pronounced aortic calcification. Although the IS rat model does not fully replicate the dialysis environment, it reinforces the mechanistic relevance of YB-1-mediated NET activation and its systemic impact on vascular pathology in uremic conditions. These findings integrate molecular and

Formatted: Shadow

experimental evidence to establish a YB-1-NET-VSMC axis that mechanistically links dyslipidemia, NET activation and VC. This mechanistic insight provides a foundation for assessing the clinical relevance of YB-1 in predicting VC risk among MHD patients.

Clinically, these mechanistic insights translate into measurable differences in serum YB-1 levels among MHD patients, providing a rationale for its application as a biomarker for VC risk. Conventional lipid markers often performed poorly in this setting due to "reverse epidemiology", where lower cholesterol levels paradoxically associate with worse outcomes (3). YB-1, integrating inflammatory and metabolic cues, demonstrated superior predictive accuracy for VC compared with traditional lipid-, glucose-, and bone-metabolism models. While ROC analysis reflects the discriminative ability of a single biomarker, DCA provided complementary evidence that YB-1 offers greater net clinical benefit within the 0.24-0.33 threshold range, corresponding to patients at intermediate predicted VC risk. This suggests that YB-1 may help refine individualized risk stratification and guide preventive interventions in MHD populations.

Strengths and limitations

This investigation is the first to uncover serum YB-1 as a novel biomarker for VC in MHD patients and uncover its mechanistic role in linking dyslipidemia to NET-mediated vascular injury. Extracellular YB-1 under uremic conditions reprograms

Formatted: Shadow

neutrophil lipid metabolism, promotes NET formation, and drives VSMC calcification. Clinically, YB-1 outperforms traditional metabolic markers, offering a novel, mechanistically informed tool for personalized VC risk stratification. Overall, these findings offer positive potential for the clinical transformation of YB-1.

However, this study has several limitations. First, VC was assessed only in coronary arteries and thoracic aorta, excluding abdominal vasculature. Second, the measurement of serum YB-1 and NETs markers at a single time point limited assessment of temporal dynamics. Third, mechanistic experiments primarily employed a gain-of-function strategy using rYB-1 without genetic silencing or neutralization. Although this approach reflects the clinical relevance of circulating YB-1, future studies using loss-of-function techniques are warranted to validate its causal role in NET formation and VC progression. Finally, although the sample size was sufficient for initial discovery, it constrains the scope of subgroup analyses and limits the generalizability of the findings.

Conclusion

This study identifies YB-1 as a promising biomarker for VC in MHD patients. It provides incremental discriminatory power beyond traditional lipid, glucose, and bone markers, and offers measurable clinical net benefit within intermediate-risk decision thresholds. Mechanistically, extracellular YB-1 enhances neutrophil lipid accumulation

Formatted: Shadow

and NET release under uremic stress, promoting VSMCs calcification. These observations point to the potential relevance of the YB-1-NET axis in refining risk stratification and guiding therapeutic strategies, aimed at reducing cardiovascular complications and improving long-term health outcomes in MHD population.

Declarations

Ethics approval and consent to participate

The study protocol received approval from the Institutional Review Board of Zhongshan Hospital (Approval No. B2021-740) and was conducted in strict accordance with the Declaration of Helsinki. Written informed consent was obtained from all participants. Animal experiments were reviewed and approved by the Institutional Animal Care and Use Committee of Fudan University (Approval No. 2023-109) and carried out in compliance with the NIH Guide for the Care and Use of Laboratory Animals.

Consent for publication

Not applicable, as this manuscript contains no identifiable information from individual participants.

Availability of data and materials

The datasets generated and analyzed during the current study are available from the corresponding authors upon reasonable request.

Formatted: Shadow

Competing interests

The authors declare no conflicts of interest, financial or otherwise, that could influence the work reported in this study.

Funding

This research was supported by the National Natural Science Foundation of China (Grant No. 82270719) and the Shanghai Natural Science Foundation (Grant No. 24ZR1411100).

Use of AI and AI-assisted Technologies Statement

No generative AI or AI-assisted tools were employed in the preparation of this manuscript.

Authors' contribution

J. Wang and X. Ding were responsible for the study conception and design. J.Wang, J. Chen, and L. Wang contributed to data collection. J. Wang, J. Chen, L. Wang, J. Cai, Z. Zhang, X. Cao, B. Shen, and Y. Fang contributed to the data analysis and interpretation. J. Chen, L. Wang, J. Cai, S. Zhao, J. Zhang, and N. Song performed *in vitro* and *in vivo* experiments. J. Wang, J. Chen, and L. Wang drafted the manuscript. J. Teng, J. Zou, and X. Ding were responsible for revising and oversighting the manuscript. All authors listed have made a substantial, direct and intellectual contributions to the work, and approved it for publication.

Acknowledgements

Formatted: Shadow

We sincerely thank the patients who participated in this study, and express our gratitude to the study personnel from the Department of Nephrology for their valuable contributions.

Clinical trial number

Not applicable.

References

1. Ahmadmehrabi S, Tang WHW. Hemodialysis-induced cardiovascular disease. Semin Dial. 2018;31:258–267.
2. Nakano T, Hiyamuta H, Yotsueda R, Tanaka S, Taniguchi M, Tsuruya K, Kitazono T. Higher cholesterol level predicts cardiovascular event and inversely associates with mortality in hemodialysis patients: 10-year outcomes of the Q-Cohort study. Ther Apher Dial. 2020;24:431–438.
3. Kalantar-Zadeh K, Block G, Humphreys MH, Kopple JD. Reverse epidemiology of cardiovascular risk factors in maintenance dialysis patients. Kidney Int. 2003;63:793–808.
4. Wanner C, Krane V, Marz W, Olschewski M, Mann J, Ruf G, Ritz E. Atorvastatin in patients with type 2 diabetes mellitus undergoing hemodialysis. N Engl J Med. 2005;353:238–248.
5. Fellstrom BC, Jardine AG, Schmieder RE, Holdaas H, Bannister K, Beutler J, Chae

Formatted: Shadow

DW, Chevaile A, Cobbe SM, Gronhagen-Riska C, Lima JJD, Lins R, Mayer G, McMahon AW, Parving H, Remuzzi G, Samuelsson O, Sonkodi S, Sci D, Sueleymanlar G, Tsakiris D, Tesar V, Todorov V, Wiecek A, Wuthrich RP, Gottlow M, Johnsson E, Zannad F. Rosuvastatin and cardiovascular events in patients undergoing hemodialysis. N Engl J Med. 2009;360:1395–1407.

6. Stenvinkel P, Carrero JJ, Axelsson J, Lindholm B, Heimburger O, Massy Z. Emerging biomarkers for evaluating cardiovascular risk in the chronic kidney disease patient: how do new pieces fit into the uremic puzzle? Clin J Am Soc Nephrol. 2008;3(2):505–521.

7. Vanholder R, Argilés A, Baurmeister U, Brunet P, Clark W, Cohen G, Deyn PPD, Deppisch R, Descamps-Latscha B, Henle T, Jorres A, Massy ZA, Rodriguez M, Stegmayr B, Stenvinkel P, Wratten ML. Uremic toxicity: present state of the art. Int J Artif Organs. 2001;24(10):695–725.

8. Josefs T, Barrett TJ, Brown EJ, Quezada A, Wu X, Voisin M, Amengual J, Fisher EA. Neutrophil extracellular traps promote macrophage inflammation and impair atherosclerosis resolution in diabetic mice. JCI Insight. 2020;5:e134796.

9. Knight JS, Luo W, O'Dell AA, Yalavarthi W, Zhao W, Subramanian V, Guo C, Grenn RC, Thompson PR, Eitzman DT, Kaplan MJ. Peptidylarginine deiminase inhibition reduces vascular damage and modulates innate immune responses in murine models of atherosclerosis. Circ Res. 2014;114(6):947–956.

Formatted: Shadow

10. Raffetseder U, Liehn EA, Weber C, Mertens PR. Role of cold shock Y-box protein-1 in inflammation, atherosclerosis and organ transplant rejection. Eur J Cell Biol. 2012;91:567–575.

11. McCauley C, Anang V, Cole B, Simmons Jr GE. Potential links between YB-1 and fatty acid synthesis in clear cell renal carcinoma. Med Res Arch. 2020;8:10.18103/mra.v8i10.2273.

12. Ma Z, Zhu Y, Wang Q, Deng M, Wang J, Li D, Gu L, Zhao R, Yan S. Y-box binding protein 1 regulates liver lipid metabolism by regulating the Wnt/□-catenin signaling pathway. Ann Transl Med. 2021;9:1693.

13. Wang J, Liu X, Gu Y, Gao Y, Jankowski V, Was N, Leitz A, Reiss LK, Shi Y, Cai J, Fang Y, Song N, Zhao S, Floege J, Ostendorf T, Ding X, Raffetseder U. DNA binding protein YB-1 is a part of the neutrophil extracellular trap mediation of kidney damage and cross-organ effects. Kidney Int. 2023;104:124–138.

14. Joint Committee for Developing Chinese Guidelines on Prevention and Treatment of Dyslipidemia in Adults. Chinese guidelines on prevention and treatment of dyslipidemia in adults (2016 revision). Chin Circ J. 2016;31(10):937–950. (in Chinese)

15. Martinuzzi E, Afonso G, Gagnerault MC, Naselli G, Mittag D, Combadiere B, Boitard C, Chaput N, Zitvogel L, Harrison LC, Mallone R. acDCs enhance human antigen-specific T-cell responses. Blood. 2011;118:2128–2137.

Formatted: Shadow

16. Cao X, Zhu N, Li L, Zhang Y, Chen Y, Zhang J, Li J, Gao C. Y-box binding protein 1 regulates ox-LDL mediated inflammatory responses and lipid uptake in macrophages. Free Radic Biol Med. 2019;141:10–20.
17. Krohn R, Raffetseder U, Bot I, Zernecke A, Shagdarsuren E, Liehn EA, Santbrink PJ, Nelson PJ, Biessen EA, Mertens PR, Weber C. Y-box binding protein-1 controls CC chemokine ligand-5 (CCL5) expression in smooth muscle cells and contributes to neointima formation in atherosclerosis-prone mice. Circulation. 2007;116:1812–1820.
18. Dhawan UK, Bhattacharya P, Narayanan S, Manickam V, Aggarwal A, Subramanian M. Hyperlipidemia impairs clearance of neutrophil extracellular traps and promotes inflammation and atherosclerotic plaque progression. Arterioscler Thromb Vasc Biol. 2021;41:2598–2615.
19. Liu Y, Carmona-Rivera C, Moore E, Seto NL, Knight JS, Pryor M, Yang ZH, Hemmers S, Remaley AT, Mowen KA, Kaplan MJ. Myeloid-specific deletion of peptidylarginine deiminase 4 mitigates atherosclerosis. Front Immunol. 2018;9:1680.
20. Molinaro R, Yu M, Sausen G, Bichsel CA, Corbo C, Folco EJ, Lee GY, Liu Y, Tesmenitsky Y, Shvartz E, Sukhova GK, Kloss F, Croce KJ, Farokhzad OC, Shi J, Libby P. Targeted delivery of protein arginine deiminase-4 inhibitors to limit arterial intimal NETosis and preserve endothelial integrity. Cardiovasc Res.

Formatted: Shadow

2021;117:2652–2663.

Formatted: Shadow

Figure Legends

Figure 1. Flowchart of participant enrollment.

Among a cohort of 303 patients undergoing MHD, 209 individuals who met the inclusion and exclusion criteria were enrolled in the final analysis.

Figure 2. Correlation between serum YB-1 and metabolic parameters in MHD patients (n=209). Scatter plots showing correlation analysis between serum YB-1 and total cholesterol **(A)**, triglycerides **(B)**, serum phosphorus **(C)**, and serum calcium **(D)**. YB-1, Y-box-binding protein-1.

Figure 3. Predictive performance and decision curve analysis of serum YB-1 and multivariable models for vascular calcification. (A) ROC curves comparing serum YB-1 (blue, AUC = 0.707) with traditional models (Model 1: AUC = 0.553; Model 2: AUC = 0.598; Model 3: AUC = 0.613). (B) The red line indicates net benefit of YB-1 alone, blue indicated Model 1 (bone-mineral), green indicates Model 2 (glucose), and purple indicates Model 3 (lipid). DCA demonstrating that serum YB-1 provided higher net clinical benefit in the threshold range of 0.24-0.33.

Figure 4. Increased NET formation and neutrophil counts in hyperlipidemia MHD patients. (A) Serum citrullinated histone H3 (citH3) levels in control (Con) and

Formatted: Shadow

hyperlipidemia (HLP) groups. (B) Absolute counts of circulating immune cell subtypes; (C) Relative distribution of lymphocyte subsets (CD3+, CD4+, CD8+, CD19+, and NK cells); group means ± SD are plotted; individual values are overlaid as open circles. *P<0.05; **P<0.01.

Figure 5. YB-1 and indoxyl sulfate promote lipid accumulation and NET formation in neutrophil-like HL-60 cells. (A-D) Relative mRNA expression of lipid metabolism-related genes: Hilpda, Srebp2, Soat1 (lipid synthesis), and ABCA1 (lipid efflux) in HL-60 cells treated with IS (1mM) and rYB-1 (100ng/ml) for 3 hours after RA-induced differentiation (n=5-9). **(E-I)** Western blot analysis and quantification of ABCA1, PGC1a, and Hilpda proteins, normalized to GAPDH. **(J)** Immunofluorescence staining showing lipid droplet accumulation (BODIPY 493/503, green) and nuclei (DAPI, blue); Scale bars, 50mm. **(K)** Western blot detection of citH3 in cell culture supernatants demonstrating increased extracellular NET-associated citH3 release upon co-stimulation with IS and rYB-1. *P<0.05; **P<0.01; ****P<0.0001.
YB-1, Y-box binding protein-1; HL-60, human promyelocytic leukemia; IS, indoxyl sulfate; rYB-1, recombinant Y-box binding protein-1.

Figure 6. NET-rich supernatants promote vascular smooth muscle cell calcification. Human VSMCs were incubated for 72 hours in osteogenic medium

Formatted: Shadow

supplemented with culture supernatants from RA-differentiated HL-60 cells pretreated with IS (1 mM) and rYB-1 (100 ng/ml). Calcium deposition was visualized using Alizarin Red S staining. Scale bars, 50mm.

Figure 7. Effects of indoxyl sulfate on lipid profile, YB-1 expression, NET formation, and vascular calcification in 5/6 nephrectomized rats. Serum creatinine **(A)**, serum lipid parameters **(B-E)**, serum YB-1 and citH3 levels **(F)** in 5/6 Nx rats treated with saline or IS for 14 weeks. **(G)** Representative images of Alizarin Red S staining in aortas showing VC. Scale bars, 100mm. **(H-K)** Relative mRNA expression of osteogenic differentiation genes: RUNX2, BMP2, BGLAP and ALPL. **P*<0.05; ***P*<0.01; *****P*<0.0001.

IS, indoxyl sulfate; 5/6 Nx, 5/6 nephrectomy; citH3, citrullinated histone H3; YB-1, Y-box binding protein-1.

Table Legends

Table 1. Clinical baseline characteristics of HD patients with and without hyperlipidemia.

ESRD, end-stage renal disease; The data in the table are expressed as mean ± standard deviation or number (%); *P < 0.05.

Formatted: Shadow

Table 2. Biological baseline characteristics of HD patients with and without hyperlipidemia.

LVEF, left ventricular ejection fraction; CIMT, carotid intima-media thickness; the data in the table are expressed as mean ± standard deviation or number (%); $*P < 0.05$; $**P < 0.01$.

Table 3. Binary logistics regression analysis to evaluate the relation between independent variables and hyperlipidemia in HD patients (N=209).

YB-1, Y-box binding protein-1; $*P < 0.05$; $**P < 0.01$.

Table 4. Performance of YB-1 and/or clinical data for predicting vascular calcification in HD patients.

YB-1, Y-box binding protein-1.

Formatted: Shadow

Serum YB-1 links dyslipidemia to NET-mediated vascular calcification in hemodialysis

ORIGINALITY REPORT

19%

SIMILARITY INDEX

PRIMARY SOURCES

1	www.mdpi.com<br>Internet	81 words — 2%
2	www.researchsquare.com<br>Internet	60 words — 1%
3	www.nature.com<br>Internet	44 words — 1%
4	"The 35th Great Wall International Congress of Cardiology Asian Heart Society Congress 2024", Cardiovascular Innovations and Applications, 2024<br>Crossref	37 words — 1%
5	public-pages-files-2025.frontiersin.org<br>Internet	30 words — 1%
6	serviciodigestivotomelloso.es<br>Internet	28 words — 1%
7	Yahao Zhang, Tingting Xiao, Jiandong Ding, Hao Jin et al. "Targeting olfactory receptor 2 on monocytes for cardioprotection against myocardial ischemia-reperfusion injury via NR4A1-mediated mitochondrial fission", Cardiovascular Research, 2025<br>Crossref	27 words — 1%

8 www.frontiersin.org
Internet
26 words — 1%

9 www.ncbi.nlm.nih.gov
Internet
25 words — 1%

10 A. Blary, E. Jenczewski. "Manipulation of crossover frequency and distribution for plant breeding", Theoretical and Applied Genetics, 2018
Crossref
24 words — 1%

11 Jialin Wang, Xiyang Liu, Yulu Gu, Yingying Gao et al. "DNA binding protein YB-1 is a part of the neutrophil extracellular trap mediation of kidney damage and cross-organ effects", Kidney International, 2023
Crossref
22 words — < 1%

12 Taffy Elian, Naufal Setiawan. "Variability and Correlation among SST, Chlorophyll-a Levels, ENSO, and Pelagic Fishing in Southern Part of Madura Strait, Indonesia, Based on Landsat 9 OLI/TIRS Imagery", Geomatics and Environmental Engineering, 2024
Crossref
19 words — < 1%

13 refubium.fu-berlin.de
Internet
19 words — < 1%

14 www.scielo.br
Internet
17 words — < 1%

15 www.science.gov
Internet
17 words — < 1%

16 ascpjournal.biomedcentral.com
Internet
14 words — < 1%

17 munin.uit.no

Internet

14 words — < 1%

18 Marc Wittmann, Jenny Dinich, Martha Merrow, Till Roenneberg. "Social Jetlag: Misalignment of Biological and Social Time", Chronobiology International, 2009
Crossref

13 words — < 1%

19 Muriel Vicent-Gil, Maria Serra-Blasco, Joan Trujols, Guillem Navarra-Ventura et al. "Cognitive discrepancy patterns and their association with psychosocial functioning across affective disorders", Spanish Journal of Psychiatry and Mental Health, 2025
Crossref

13 words — < 1%

20 docksci.com
Internet

13 words — < 1%

21 irakleitos2.aua.gr
Internet

13 words — < 1%

22 panafrican-med-journal.com
Internet

13 words — < 1%

23 pubmed.ncbi.nlm.nih.gov
Internet

13 words — < 1%

24 James Walker, Jingyi Zhang, Yalin Liu, Shujuan Xu et al. "Extensive N4 cytosine methylation is essential for Marchantia sperm function", Cell, 2025
Crossref

12 words — < 1%

25 Shiun-Yuan Hsu, Shao-Chun Wu, Cheng-Shyuan Rau, Ting-Min Hsieh et al. "Impact of Adapting the Abbreviated Injury Scale (AIS)-2005 from AIS-1998 on Injury Severity Scores and Clinical Outcome", International Journal of Environmental Research and Public Health, 2019
Crossref

12 words — < 1%

26	assets-eu.researchsquare.com<br>Internet	12 words — < 1%
27	kipdf.com<br>Internet	12 words — < 1%
28	mdpi-res.com<br>Internet	12 words — < 1%
29	eurjmedres.biomedcentral.com<br>Internet	11 words — < 1%
30	www.esrs.eu<br>Internet	11 words — < 1%
31	www.fedoa.unina.it<br>Internet	11 words — < 1%
32	Diana A. Odhiambo, Allison N. Pittman, Ashlyn G. Rickard, Rico J. Castillo et al. "Preclinical Evaluation of the ATR Inhibitor BAY 1895344 as a Radiosensitizer for Head and Neck Squamous Cell Carcinoma", International Journal of Radiation Oncology*Biology*Physics, 2023<br>Crossref	10 words — < 1%
33	Franciel Batista Felix, Jessica Maria Dantas Araújo, Elindayane Vieira de Souza, Vanessa Pinho et al. "Biochanin A attenuates zymosan-induced arthritis in mice similarly to 17-β estradiol: an alternative to hormone replacement therapy?", Inflammation Research, 2020<br>Crossref	10 words — < 1%
34	Herencia, Carmen, Mª Encarnación Rodríguez-Ortiz, Juan R. Muñoz-Castañeda, Julio Manuel Martinez-Moreno, Rocío Canalejo, Addy Montes de Oca, Juan	10 words — < 1%

M. Díaz-Tocados, Esther Peralbo-Santaella, Carmen Marín, Antonio Canalejo, Mariano Rodriguez, and Yolanda Almaden. "Angiotensin II prevents calcification in vascular smooth muscle cells by enhancing magnesium influx", European Journal of Clinical Investigation, 2015.
Crossref

35 cjasn.asnjournals.org
Internet
10 words — < 1%

36 link.springer.com
Internet
10 words — < 1%

37 www.wjgnet.com
Internet
10 words — < 1%

38 Eric Schiffer. "Markers of vascular disease in plasma from patients with chronic kidney disease identified by proteomic analysis :", Journal of Hypertension, 04/2011
Crossref
9 words — < 1%

39 Tinghang Yang, Jing Peng, Zhuyun Zhang, Yu Chen, Zhihui Liu, Luojia Jiang, Lunqiang Jin, Mei Han, Baihai Su, Yupei Li. "Emerging therapeutic strategies targeting extracellular histones for critical and inflammatory diseases: an updated narrative review", Frontiers in Immunology, 2024
Crossref
9 words — < 1%

40 Yalavarthi, Srilakshmi, Travis J. Gould, Ashish N. Rao, Levi F. Mazza, Alexandra E. Morris, Carlos Núñez-Álvarez, Diego Hernández-Ramírez, Paula L. Bockenstedt, Patricia C. Liaw, Antonio R. Cabral, and Jason S. Knight. "Antiphospholipid antibodies promote the release of neutrophil extracellular traps: A new mechanism of thrombosis in the antiphospholipid syndrome", Arthritis & Rheumatology, 2015.
9 words — < 1%

Crossref

41	bmcgeriatr.biomedcentral.com<br>Internet	9 words — < 1%
42	www.kijob.or.kr<br>Internet	9 words — < 1%
43	Jovana Kaludjerovic, Hirotaka Komaba, Tadatoshi Sato, Reinhold G. Erben et al. "Klotho expression in long bones regulates FGF23 production during renal failure", The FASEB Journal, 2017<br>Crossref	8 words — < 1%
44	Nigel D TOUSSAINT. "Using vertebral bone densitometry to determine aortic calcification in patients with chronic kidney disease", Nephrology, 02/2010<br>Crossref	8 words — < 1%
45	Yangtian Yan, Ningning Yuan, Yuchi Chen, Yun Ma et al. "SKP alleviates the ferroptosis in diabetic kidney disease through suppression of HIF-1α/HO-1 pathway based on network pharmacology analysis and experimental validation.", Research Square Platform LLC, 2023<br>Crossref Posted Content	8 words — < 1%
46	Yuanyuan Liu, Jing Liu, Shuqi Hou, Lingling Chang, Hanli Wu. "The Impact of Frailty on the Survival Prognosis of Maintenance Hemodialysis Patients and the Construction and Validation of a Survival Prediction Model", Springer Science and Business Media LLC, 2025<br>Crossref Posted Content	8 words — < 1%
47	www.biorxiv.org<br>Internet	8 words — < 1%
48	www.imrpress.com<br>Internet	8 words — < 1%

49 Dennis C. Ang, Kathleen Thomas, Kurt Kroenke. "An Exploratory Study of Primary Care Physician Decision Making Regarding Total Joint Arthroplasty", Journal of General Internal Medicine, 2007 — 7 words — < 1%
Crossref

50 Mohamed Moinuddin, Mohammed Mohsin Zia. "Characterisation of Peritoneal Calcification in Encapsulating Peritoneal Sclerosis", The University of Manchester (United Kingdom), 2025 — 7 words — < 1%
ProQuest

51 Shixing Zheng, Liudmila Matskova, Xiaoying Zhou, Xue Xiao, Guangwu Huang, Zhe Zhang, Ingemar Ernberg. "Downregulation of adipose triglyceride lipase by EB viral-encoded LMP2A links lipid accumulation to increased migration in nasopharyngeal carcinoma", Molecular Oncology, 2020 — 7 words — < 1%
Crossref

52 Shuai Yu, Yaya Ren, Jiangang Song, Yuxin Zhu, Hua Jiang, Yuanxia Li. "Application of Explainable Machine Learning in Early Diagnosis Models for Risk Prediction of Severe Mycoplasma pneumoniae Pneumonia in Children", Springer Science and Business Media LLC, 2025 — 6 words — < 1%
Crossref Posted Content

EXCLUDE QUOTES	ON	EXCLUDE SOURCES	OFF
EXCLUDE BIBLIOGRAPHY	ON	EXCLUDE MATCHES	OFF
